# Supplementary material for: Epigenetic modulation reveals differentiation state specificity of oncogene addiction
Source: Nat Commun. 2021 Mar 9;12:1536. doi: 10.1038/s41467-021-21784-2 (PMC7943789; doi:10.1038/s41467-021-21784-2)
Supplement: Supplementary file 1 — Supplementary Information [file 41467_2021_21784_MOESM1_ESM.pdf]

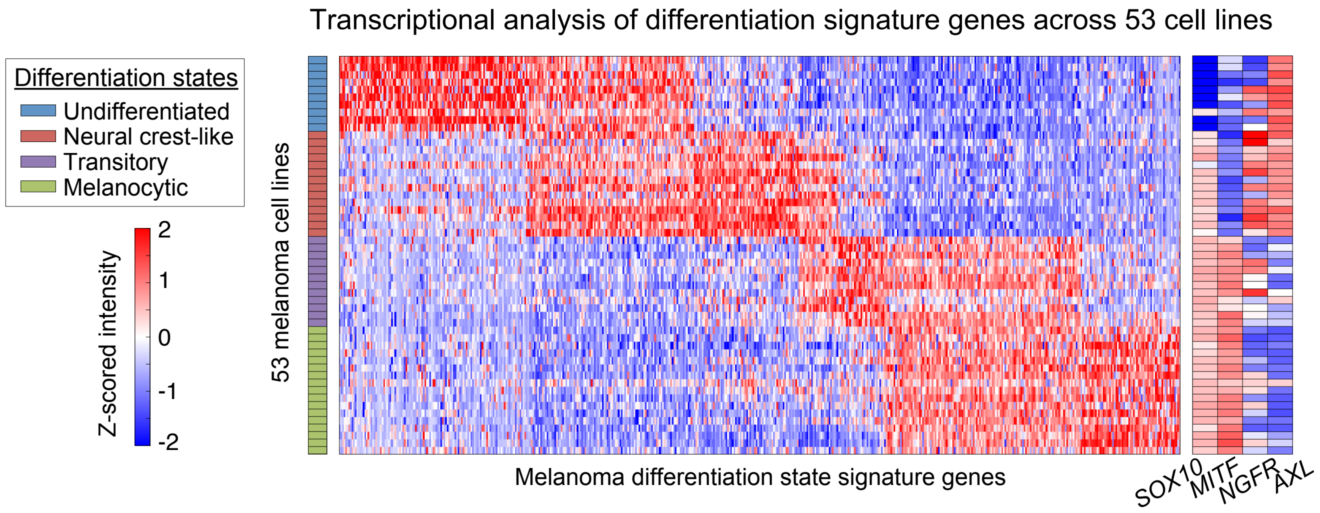

Supplementary Figure 1. Four melanoma differentiation subtypes revealed by transcriptomic analysis of 53 patient-derived melanoma cell lines from Tsoi *et al*, Cancer Cell 33, 2018. These states can be distinguished by differentiation state markers *SOX10*, *MITF*, *NGFR* and *AXL*. Source data are provided as a Source Data file.

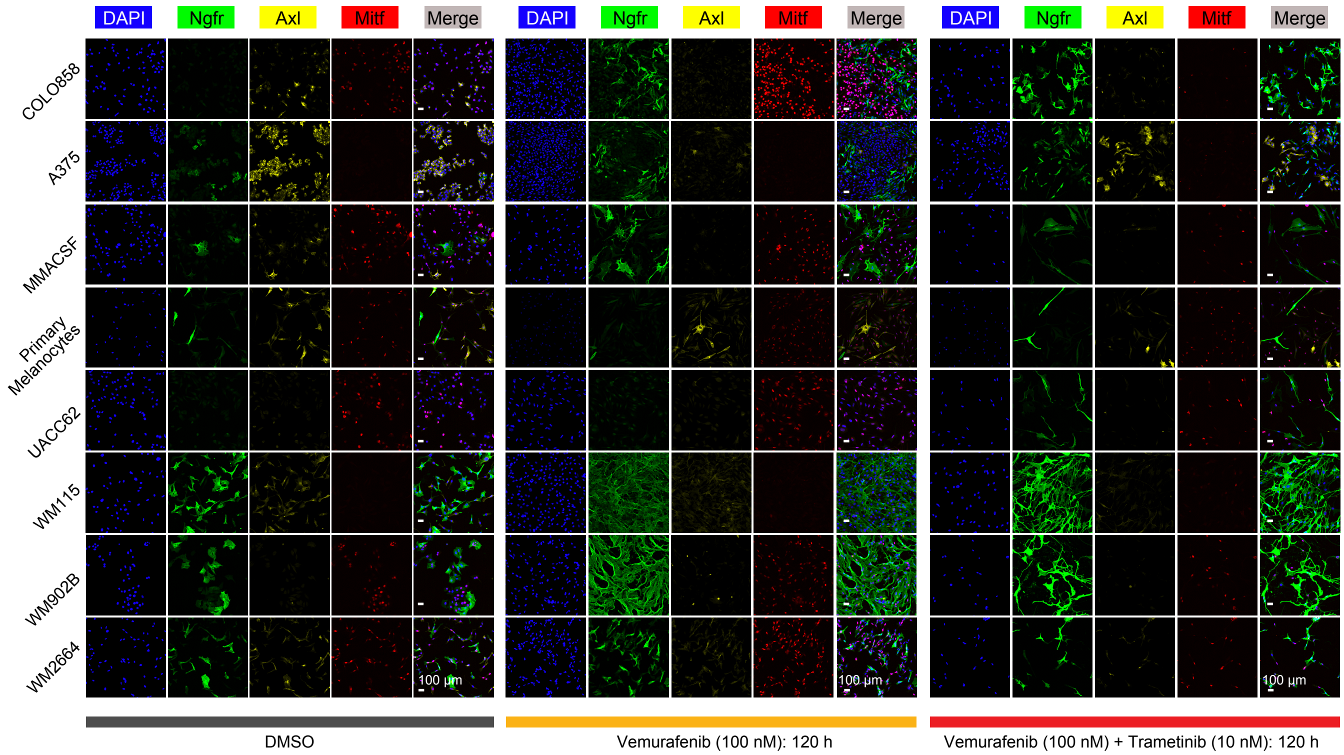

Supplementary Figure 2. Representative multiplexed immunofluorescence images of differentiation state markers Mitf, Ngfr and Axl, co-stained in indicated melanoma cell lines or primary melanocytes before and after treatment with Braf/Mek inhibitors at indicated doses and timepoints. Each experiment was repeated twice independently with similar result. Scale bars represent 100 µm.

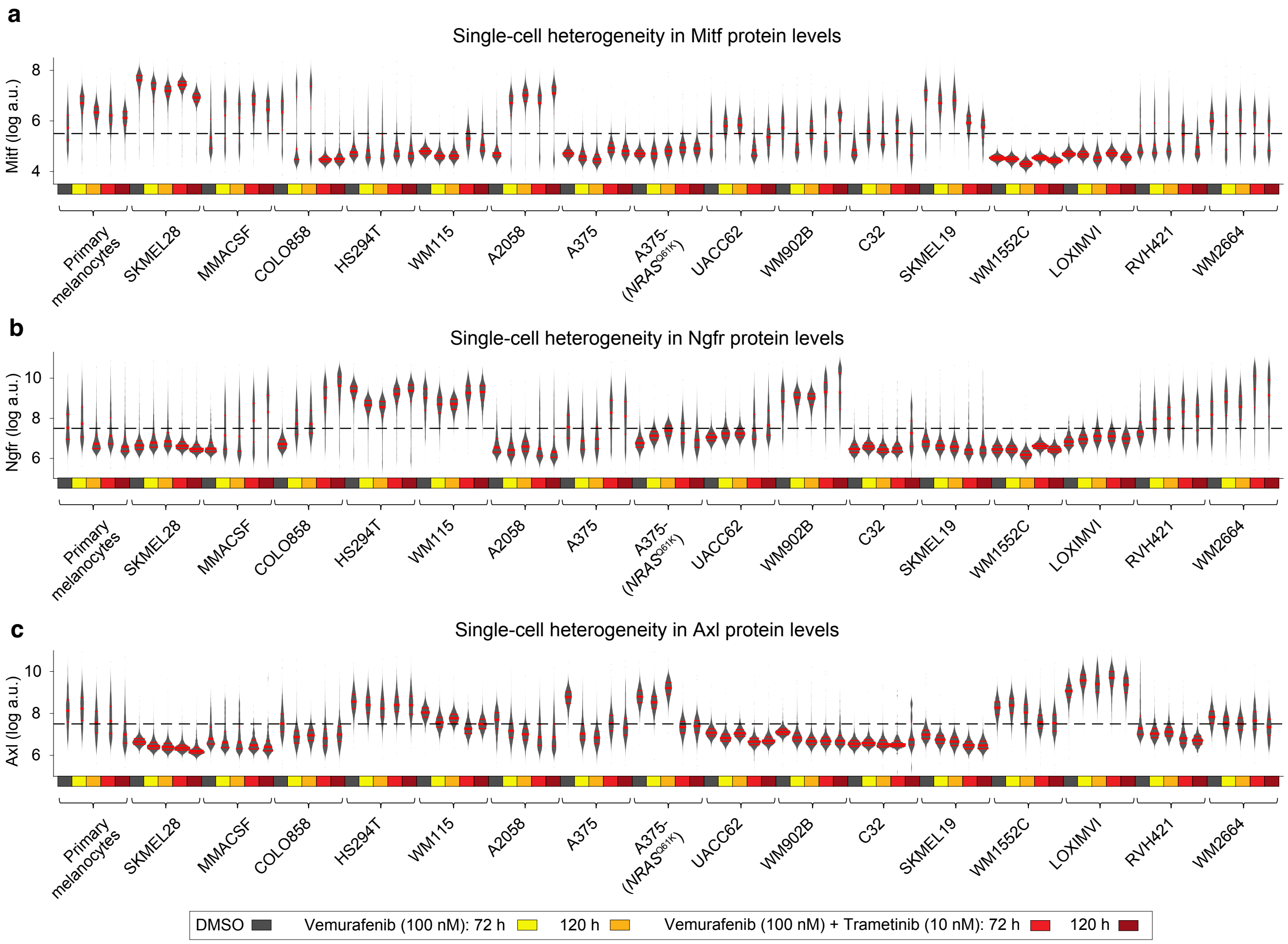

Supplementary Figure 3. Single-cell heterogeneity in differentiation state markers Mitf, Ngfr and Axl, quantified by multiplexed immunofluorescence imaging across 16 melanoma cell lines and primary melanocytes before and after treatment with Braf/Mek inhibitors at indicated doses and timepoints. The distributions of single-cell data across different conditions are shown by violin plots, highlighting the median and interquartile (25% and 75%) ranges. Source data are provided as a Source Data file.

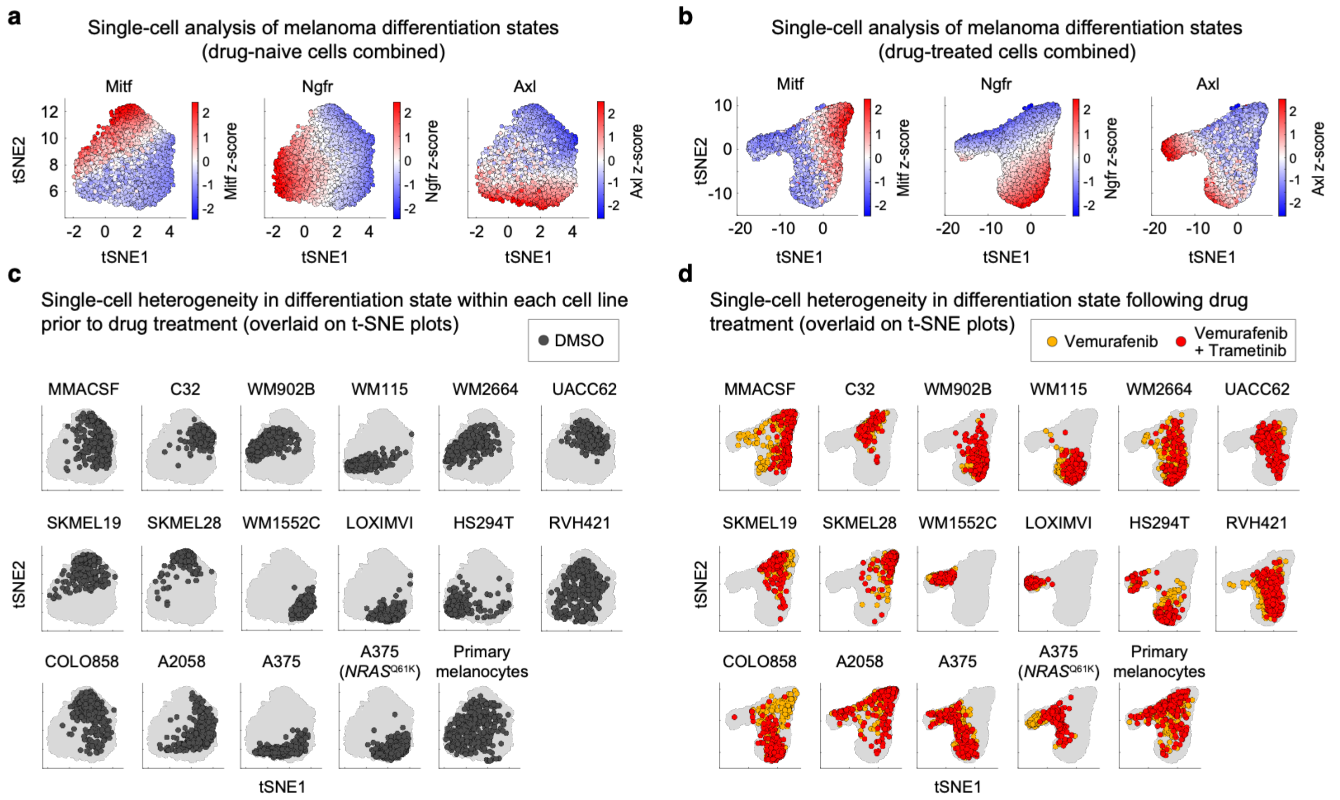

Supplementary Figure 4. Single-cell analysis uncovers heterogeneities in melanoma differentiation, proliferation and MAPK signaling states. (a,b) Single-cell protein levels of three melanoma differentiation state markers, Mitf, Ngfr and Axl, measured by multiplexed immunofluorescence microscopy and visualized by t-SNE, performed separately for drug-naïve cells **(a)** and Braf/Mek inhibitor-treated cells **(b)**. Experimental conditions and cells utilized for this analysis are the same as those depicted in Figure 1. **(c,d)** Projections of single-cell variations within each individual cell line on t-SNE maps. Source data are provided as a Source Data file.

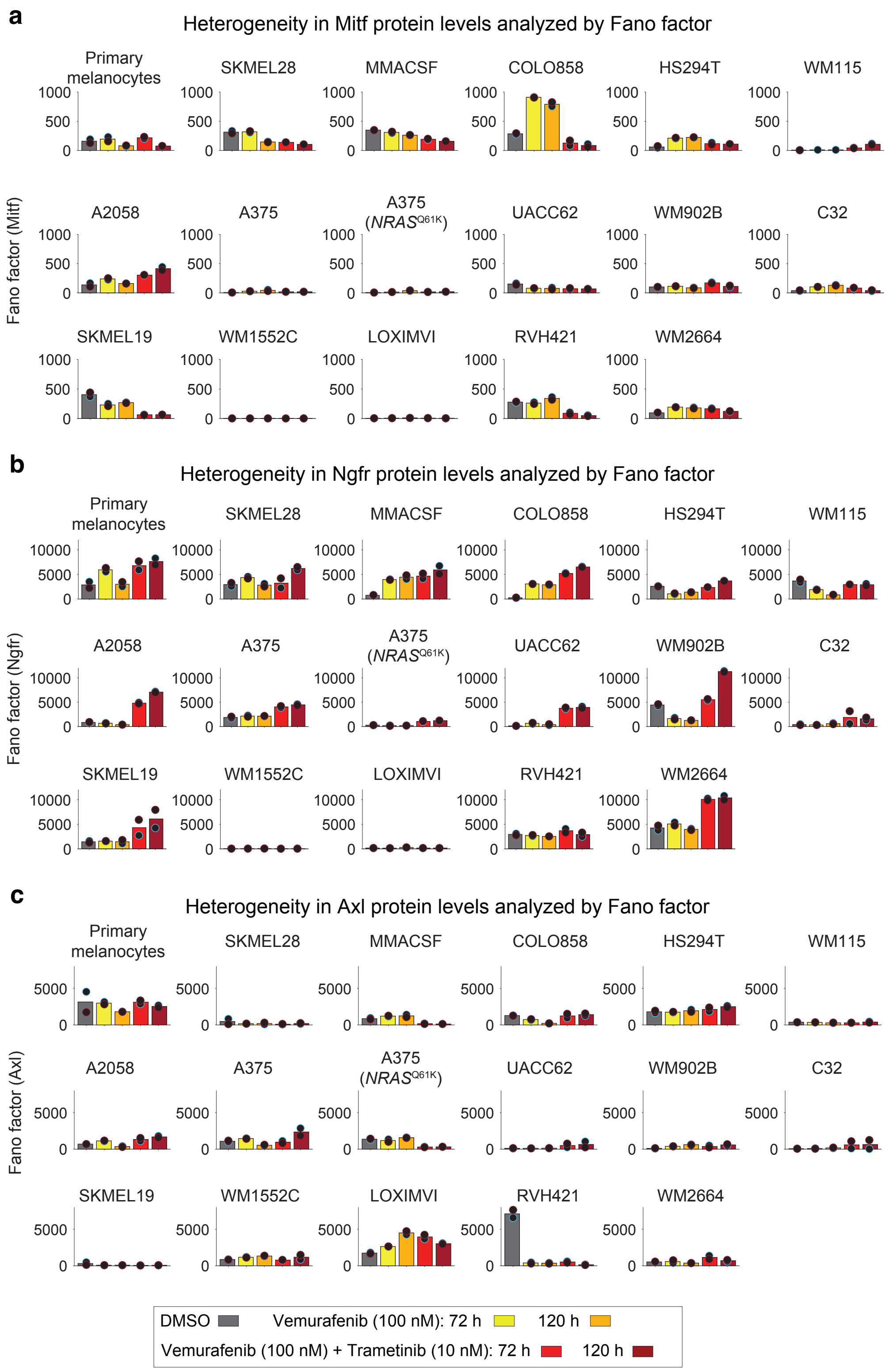

Supplementary Figure 5. Heterogeneity in differentiation state markers Mitf, Ngfr and Axl, revealed by Fano factor analysis of primary melanocytes and 16 melanoma cell lines treated with Braf/Mek inhibitors at indicated doses and timepoints. Data are presented as mean of n = 2 biologically independent experiments. Source data are provided as a Source Data file.

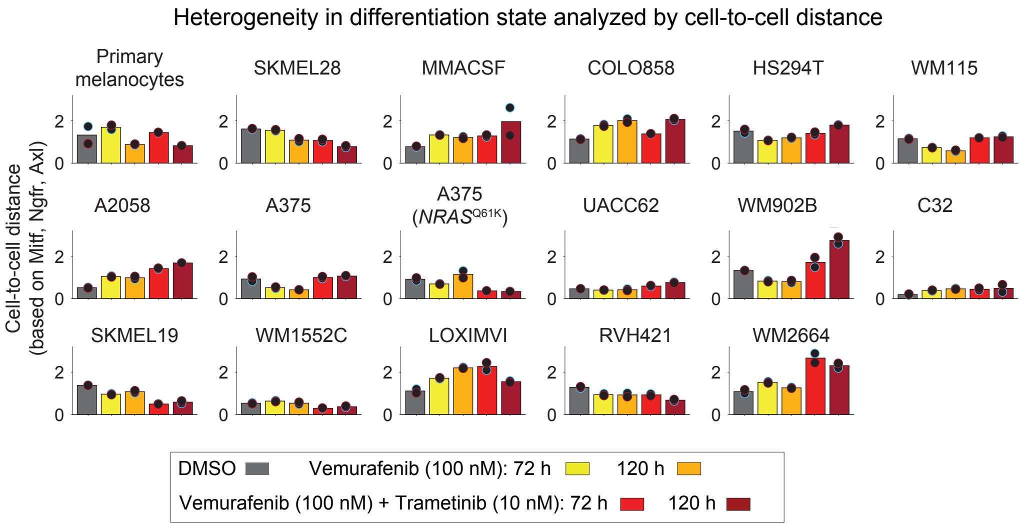

Supplementary Figure 6. Heterogeneity in differentiation state markers Mitf, Ngfr and Axl, revealed by cell-to-cell distance analysis of primary melanocytes and 16 melanoma cell lines co-stained simultaneously for all three protein markers following treatment with Braf/Mek inhibitors at indicated doses and timepoints. Data are presented as mean of n = 2 biologically independent experiments. Source data are provided as a Source Data file.

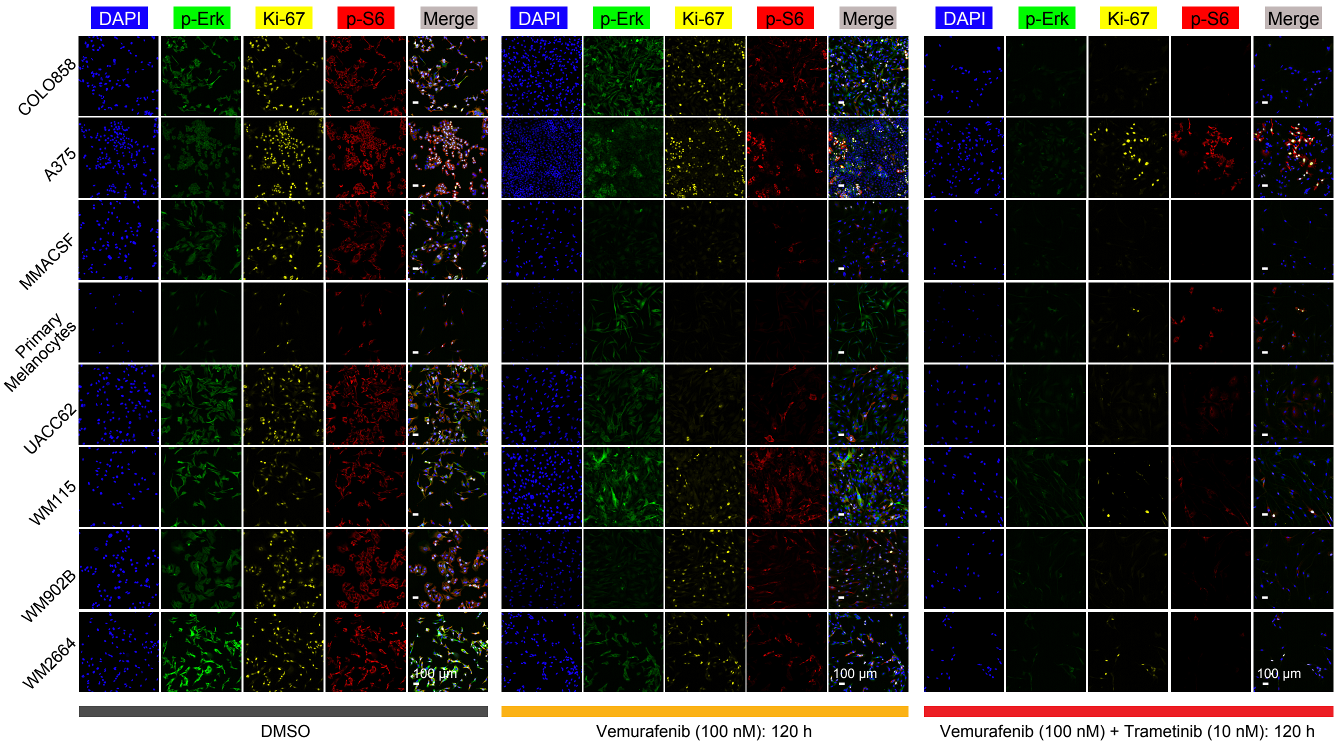

Supplementary Figure 7. Representative multiplexed immunofluorescence images of p-Erk$^{T202/Y204}$, p-S6$^{S235/S236}$ and Ki-67 proteins, co-stained in indicated melanoma cell lines or primary melanocytes before and after treatment with Braf/Mek inhibitors at indicated doses and timepoints. Each experiment was repeated twice independently with similar result. Scale bars represent 100 µm.

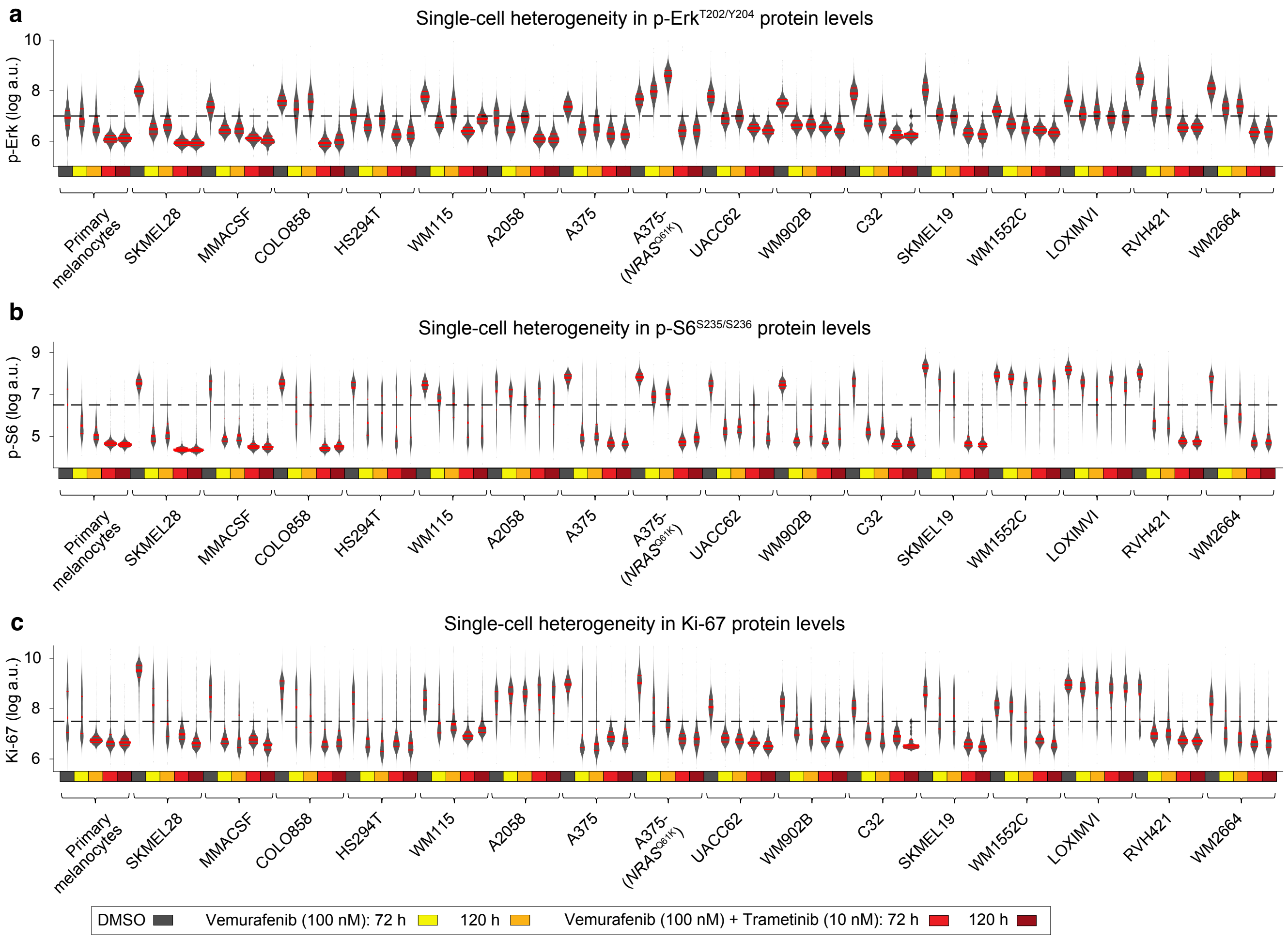

Supplementary Figure 8. Single-cell heterogeneity in p-Erk$^{T202/Y204}$, p-S6$^{S235/S236}$, and Ki-67 proteins, quantified by multiplexed immunofluorescence imaging across 16 melanoma cell lines and primary melanocytes before and after treatment with Braf/Mek inhibitors at indicated doses and timepoints. The distributions of single-cell data across different conditions are shown by violin plots, highlighting the median and interquartile (25% and 75%) ranges. Source data are provided as a Source Data file.

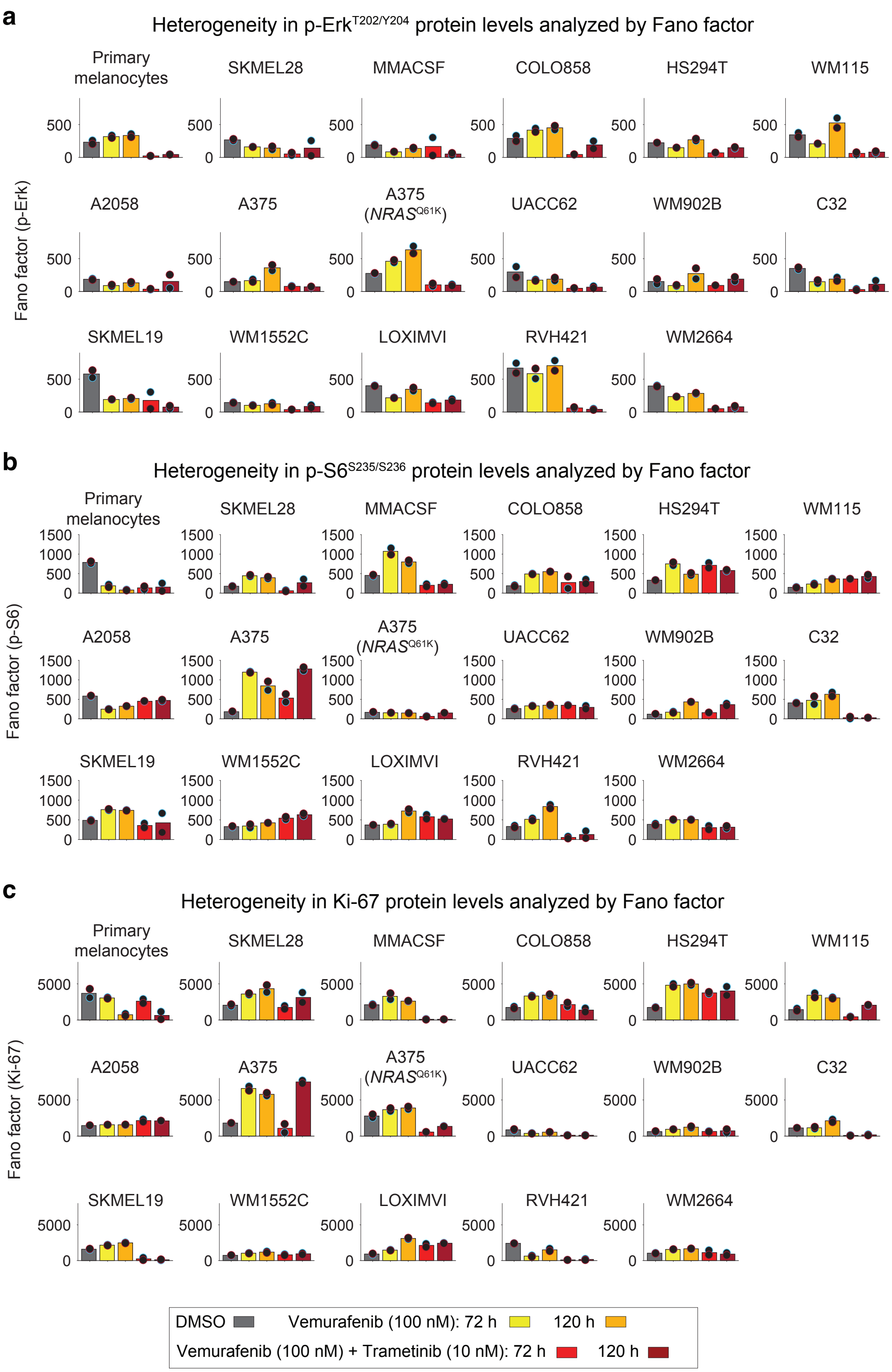

Supplementary Figure 9. Heterogeneity in p-Erk$^{T202/Y204}$, p-S6$^{S235/S236}$ and Ki-67 proteins, revealed by Fano factor analysis of primary melanocytes and 16 melanoma cell lines treated with Braf/Mek inhibitors at indicated doses and timepoints. Data are presented as mean of n = 2 biologically independent experiments. Source data are provided as a Source Data file.

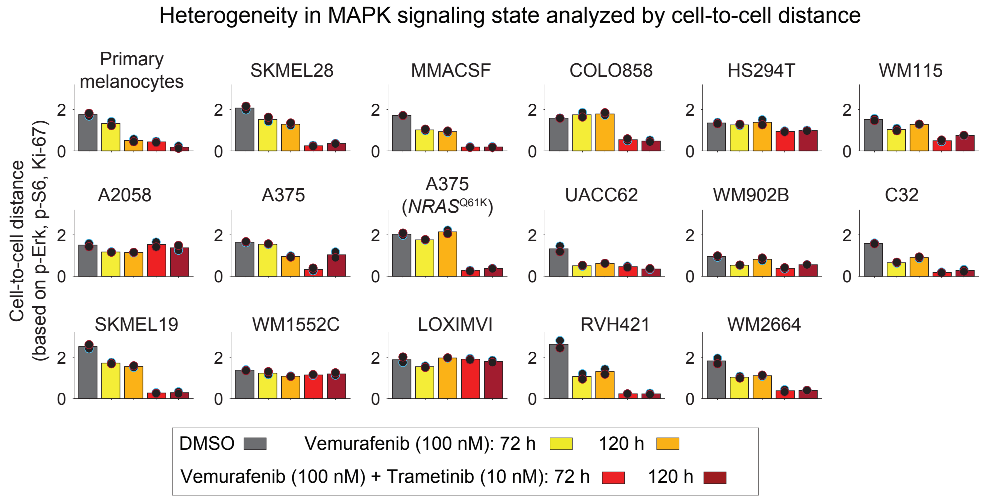

Supplementary Figure 10. Heterogeneity in p-Erk$^{T202/Y204}$, p-S6$^{S235/S236}$ and Ki-67 proteins, revealed by cell-to-cell distance analysis of primary melanocytes and 16 melanoma cell lines co-stained simultaneously for all three protein markers following treatment with Braf/Mek inhibitors at indicated doses and timepoints. Data are presented as mean of n = 2 biologically independent experiments. Source data are provided as a Source Data file.

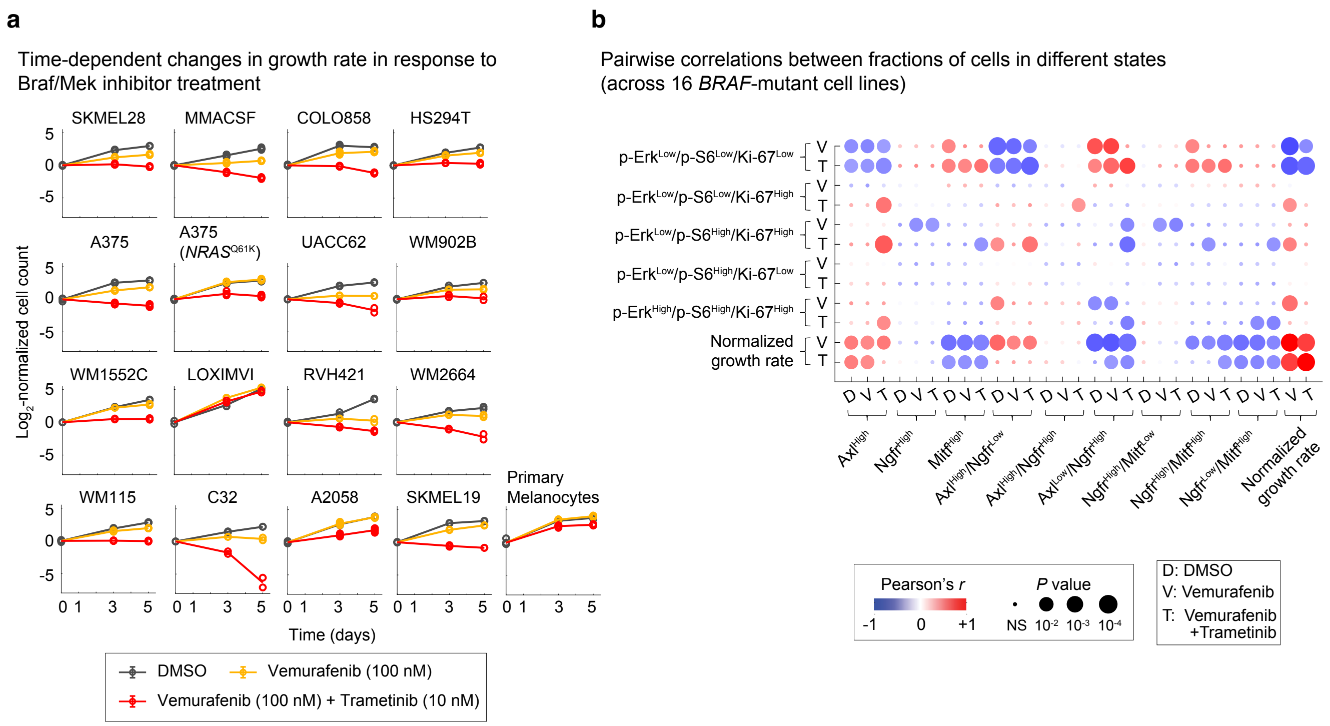

Supplementary Figure 11. Single-cell analysis uncovers heterogeneities in melanoma differentiation, proliferation and MAPK signaling states across a wide range of Braf/Mek inhibitor sensitivity. (a) Log_2-normalized changes in live cell count across three timepoints (including 0, 3, and 5 days) following exposure of 16 *BRAF*$^{V600E/D}$ melanoma cell lines and primary melanocytes to DMSO, vemurafenib (at 100 nM) or vemurafenib (at 100 nM) plus trametinib (at 10 nM). Data are presented as the average of n = 2 biologically independent samples. **(b)** Two-sided Pearson's correlation analysis between diverse differentiation states, MAPK signaling states, and normalized growth rates cross 16 melanoma cell lines treated with Braf inhibitor (vemurafenib at 100 nM) or the combination of Braf and Mek inhibitors (vemurafenib at 100 nM and trametinib at 10 nM) for 3-5 days. Source data are provided as a Source Data file.

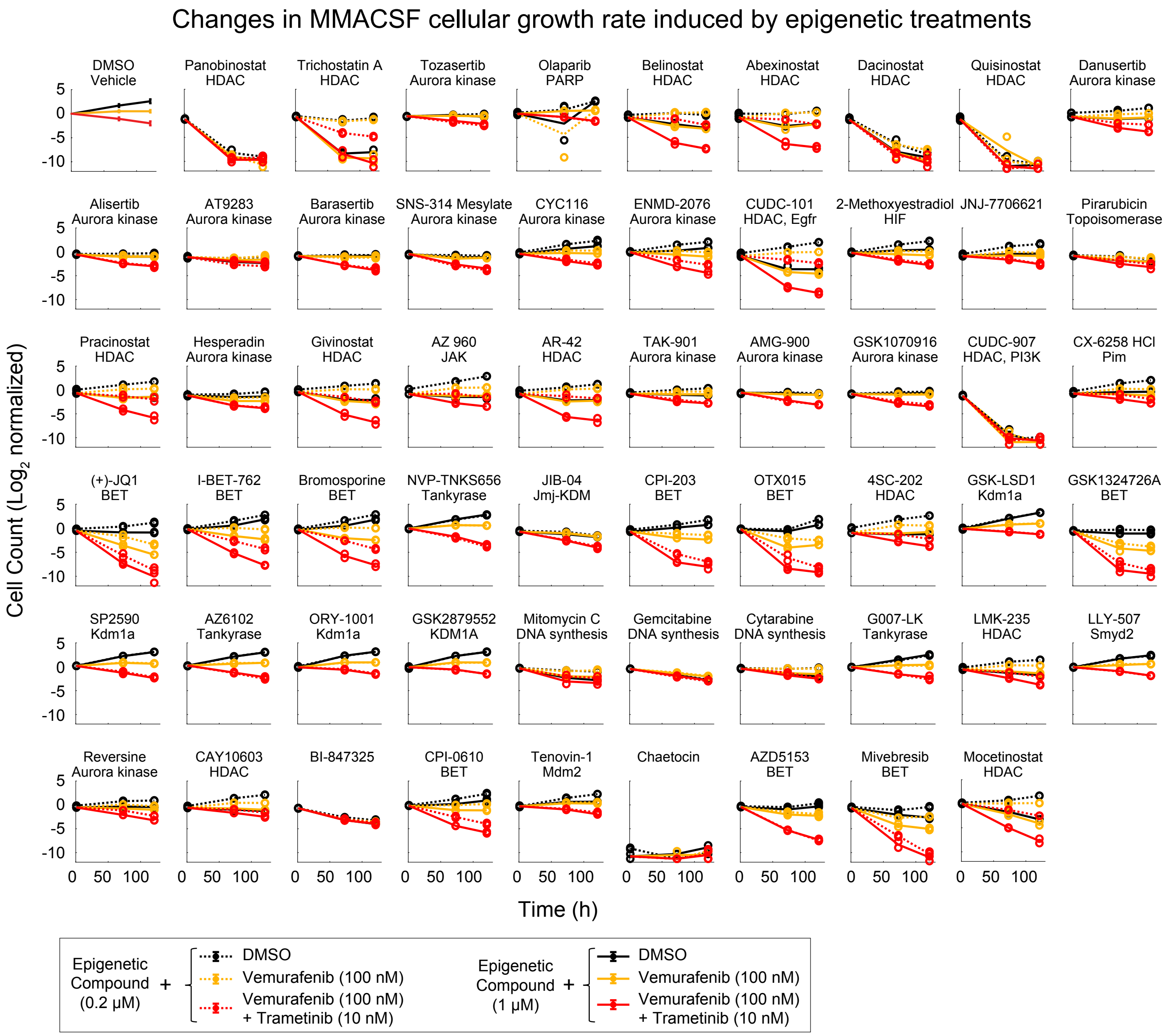

Supplementary Figure 12. Changes in MMACSF cellular growth rate induced by significant epigenetic treatments. Log_2-normalized changes in live cell count following exposure of MMACSF cells (pretreated for 24 h with either DMSO or two different doses of 58 significant epigenetic compounds) to either DMSO, vemurafenib (at 100 nM), or vemurafenib (at 100 nM) plus trametinib (at 10 nM), for a period of 3-5 days. Individual compounds and their nominal epigenetic targets are indicated. Data are presented as mean of n = 2 biologically independent experiments. Source data are provided as a Source Data file.

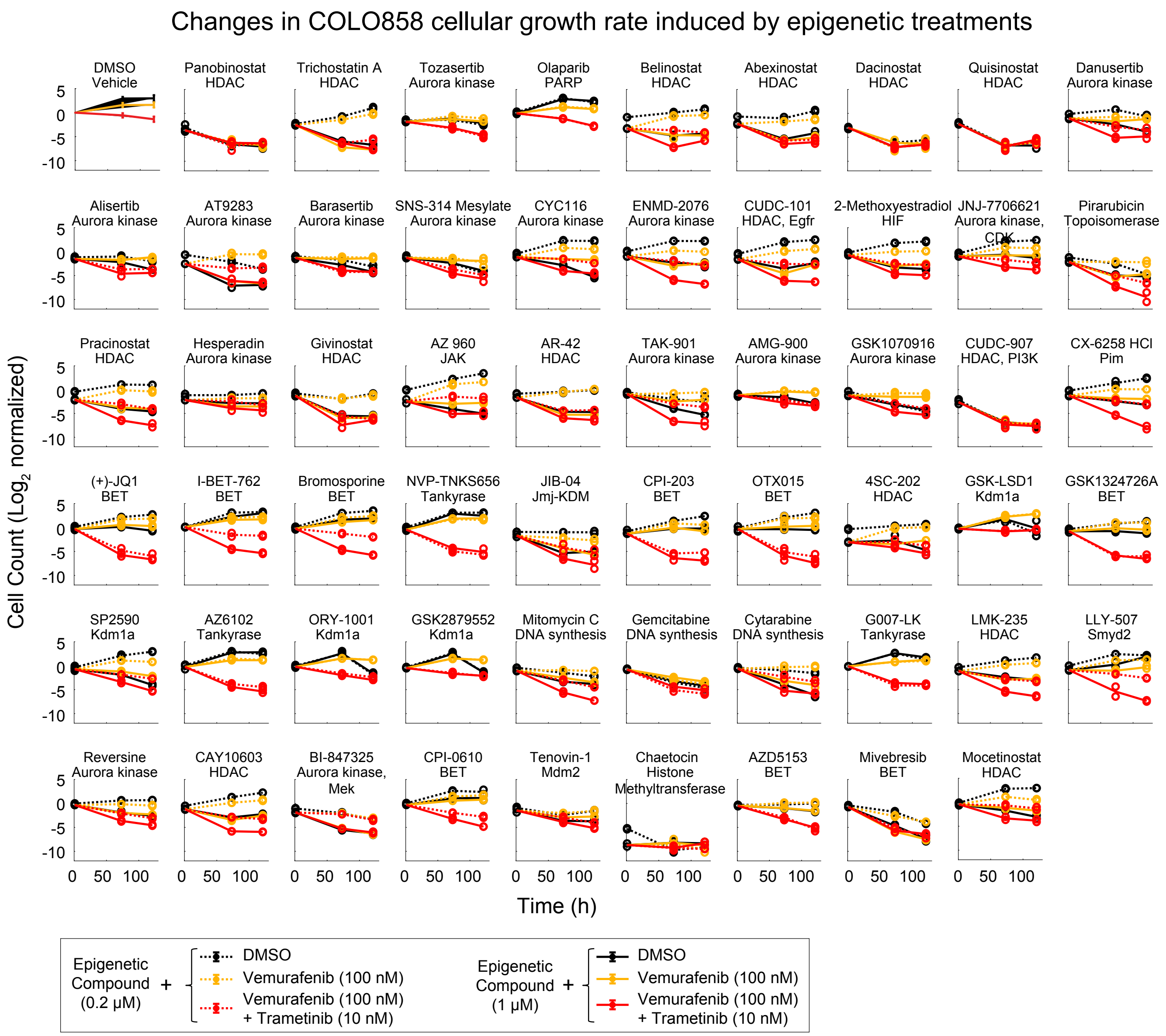

Supplementary Figure 13. Changes in COLO858 cellular growth rate induced by significant epigenetic treatments. Log_2-normalized changes in live cell count following exposure of COLO858 cells (pretreated for 24 h with either DMSO or two different doses of 58 significant epigenetic compounds) to either DMSO, vemurafenib (at 100 nM), or vemurafenib (at 100 nM) plus trametinib (at 10 nM), for a period of 3-5 days. Individual compounds and their nominal epigenetic targets are indicated. Data are presented as mean of n = 2 biologically independent experiments. Source data are provided as a Source Data file.

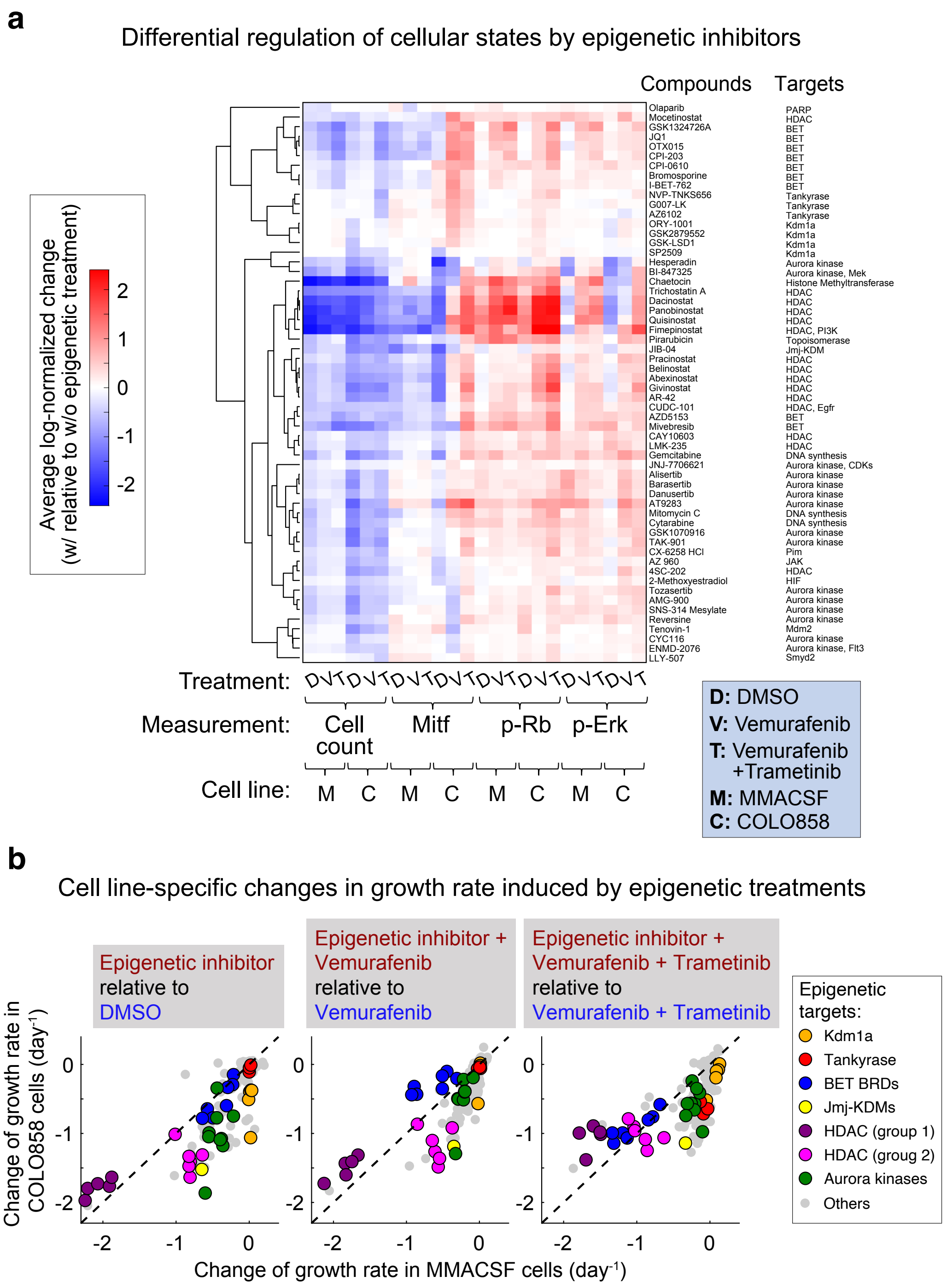

Supplementary Figure 14. Differential regulation of cellular states by mechanistically distinct epigenetic-modifying compounds. (a) Hierarchical clustering of normalized measurements of cellular grow rate, Mitf, p-Rb$^{S807/811}$, and p-Erk$^{T202/Y204}$ at indicated treatment conditions and timepoints in MMACSF and COLO858 cells as described in Figure 2b. **(b)** Cell line-specific changes in growth rate induced by each class of epigenetic treatments. Growth rate data collected for cells treated with each epigenetic compound and MAPK inhibitor condition (i.e., DMSO, vemurafenib, and vemurafenib plus trametinib) were normalized to cells treated without any epigenetic compound and the same MAPK inhibitor condition. Source data are provided as a Source Data file.

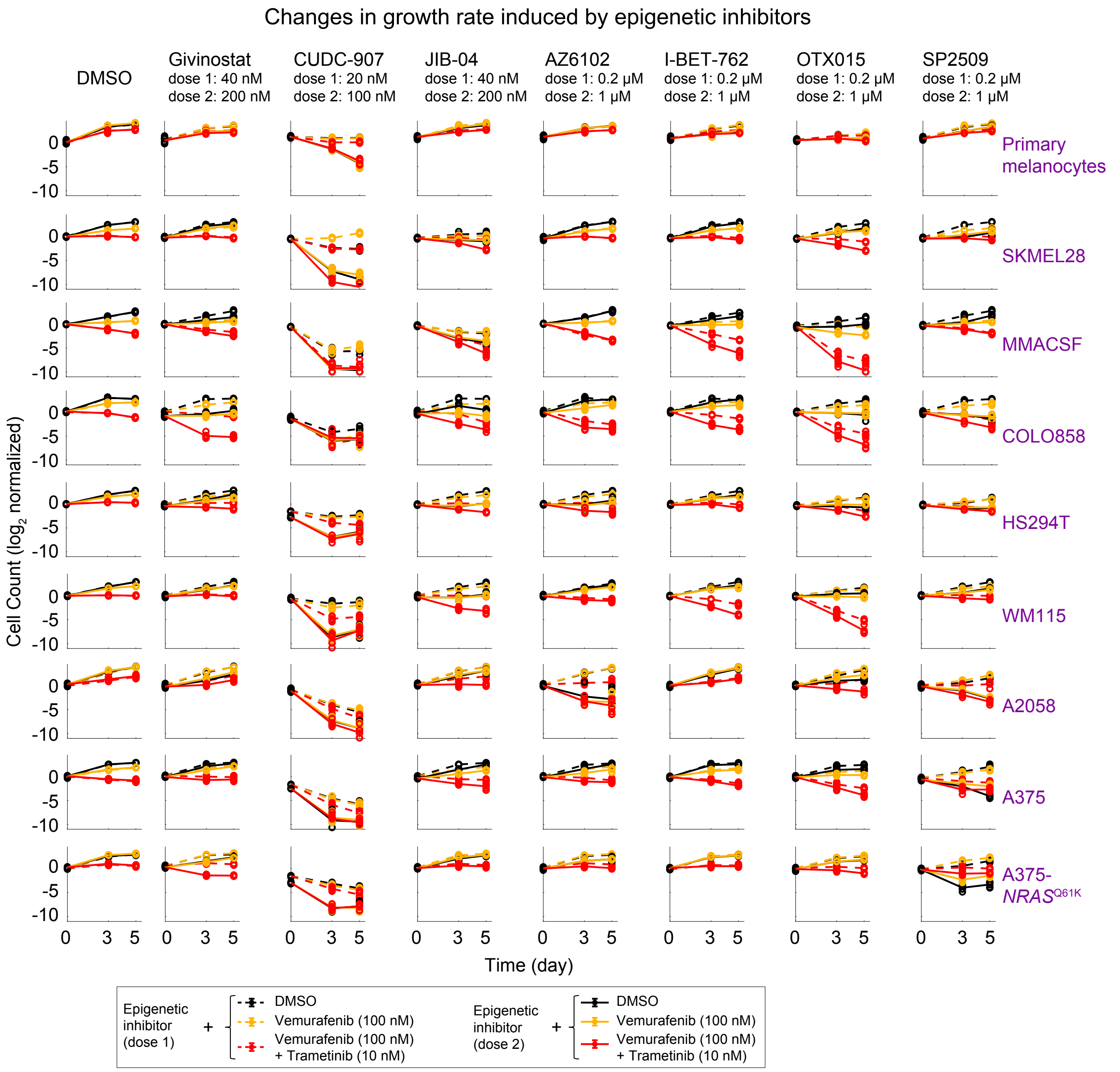

Supplementary Figure 15. Epigenetic treatment-induced changes in growth rates of 8 melanoma cell lines and primary melanocytes to identify optimized drug doses. Log_2-normalized changes in live cell count following exposure of eight different melanoma cell lines and non-transformed primary melanocytes (pretreated for 24 h with either DMSO or two different doses of seven different epigenetic compounds) to either DMSO, vemurafenib (at 100 nM), or vemurafenib (at 100 nM) plus trametinib (at 10 nM), for a period of 3-5 days. Treatment doses for each compound are as follows: Givinostat (40 and 200 nM), CUDC-907 (20 and 100 nM), JIB-04 (40 and 200 nM), AZ6102 (0.2 and 1 μM), I-BET-762 (0.2 and 1 μM), OTX015 (0.2 and 1 μM), SP2509 (0.2 and 1 μM). Data are presented as mean of n = 6 biologically independent samples. Source data are provided as a Source Data file.

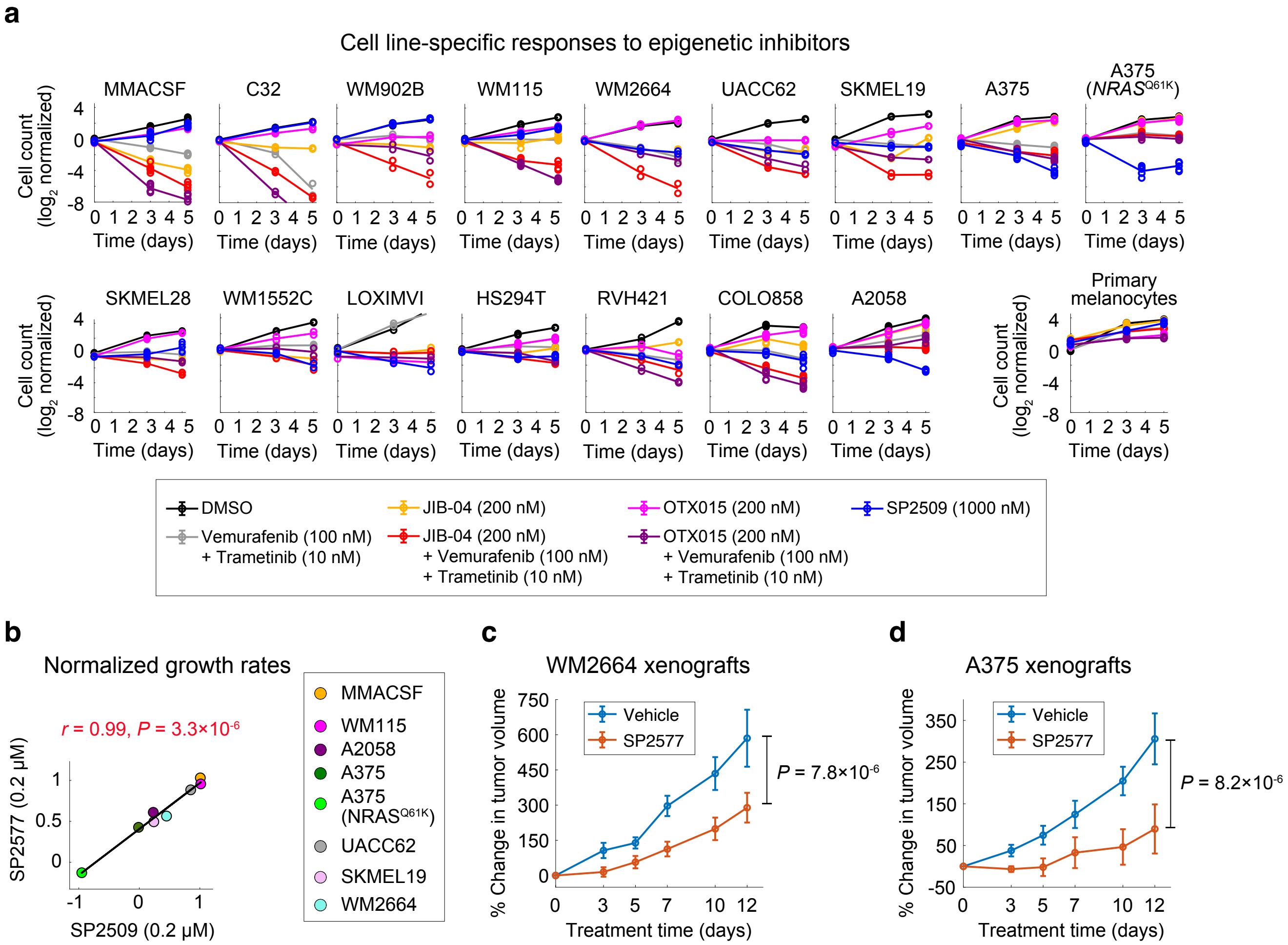

Supplementary Figure 16. Comparing treatment-induced changes in growth rates of melanoma cells following pharmacological inhibition of Kdm1a, Jmj-KDMs and BET proteins. (a) Log_2-normalized changes in live cell count following exposure of melanoma cell lines and non-transformed primary melanocytes to different drugs at indicated doses for a period of 5 days. Data are presented as the average of n = 6 biologically independent samples (in case of MMACSF, WM115, HS294T, A2058, A375, A375(*NRAS*^{Q61K}), SKMEL28, COLO858 and primary melanocytes), or the average of n = 2 biologically independent samples (in case of C32, WM2664, UACC62, SKMEL19, WM902B, WM1552C, LOXIMVI, RVH421). **(b)** Two-sided Pearson's correlation between the effect (i.e. normalized growth rates) of Kdm1a inhibitor SP2509 (at 0.2 µM) and its clinical formulation, SP2577 (at 0.2 µM) across eight *BRAF*-mutant melanoma cell lines. **(c,d)** Mice bearing xenografts of WM2664 cells (**c**) and A375 cells (**d**) were treated as shown by the Kdm1a inhibitor SP2577 or vehicle for a period of 12 days, to determine the effect on tumor growth. Data represent mean values ± s.e.m across 5 mice per group. Statistical significance was determined by two-way ANOVA. Source data are provided as a Source Data file.

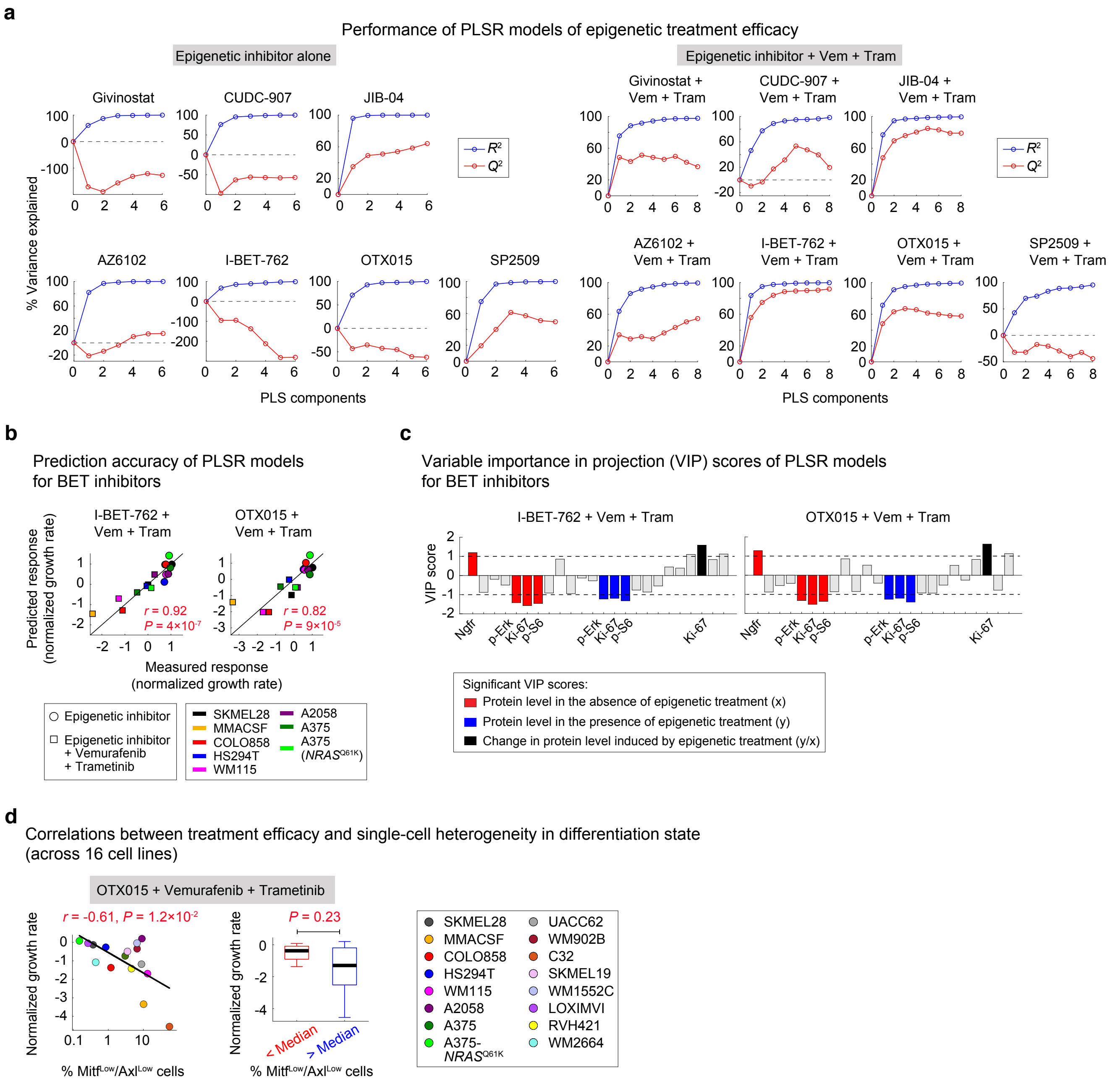

Supplementary Figure 17. Multivariate modeling identifies differentiation state-specific predictors of epigenetic inhibitor efficacy. (a) Partial least square regression (PLSR) model calibration for the effect of each of the seven epigenetic inhibitors, used individually (left panels) or in combination with vemurafenib and trametinib (right panels), across eight *BRAF*-mutant melanoma cell lines. R^2 values (representing goodness of fit) and Q^2 values (representing prediction accuracy based on leave-one-out cross-validation) are shown for models built with increasing numbers of PLS components. **(b)** Two-sided Pearson's correlation between responses (normalized growth rates) to I-BET-762 (left) or OTX015 (right) in combination with vemurafenib and trametinib measured for each of the eight melanoma cell lines (x-axis) and corresponding responses predicted by PLSR modeling following leave-one-out cross validation (y-axis). **(c)** PLSR-derived variable importance in the projection (VIP) scores highlighting combinations of protein measurements at the baseline (shown in red), following epigenetic inhibitor

treatment (shown in blue), or the ratio of change induced by each epigenetic compound (shown in black), that are predictive of efficacy for I-BET-762 or OTX015 in combination with vemurafenib and trametinib. The sign of VIP score shows whether the change in variable correlated negatively or positively with treatment-induced response. Only VIP scores of greater than 1 or smaller than -1 with a statistically significant two-sided Pearson's correlation ($P < 0.05$) are highlighted. **(d)** Association of epigenetic compound efficacy with baseline melanoma differentiation state characterized at a single-cell level across sixteen *BRAF*-mutant cell lines. The significance of differences between normalized growth rate in response to OTX015 combined with vemurafenib and trametinib versus the baseline fraction of $Mitf^{Low}/Axl^{Low}$ cells was evaluated by *P* values based on two-sided Pearson's correlation analysis (left) and two-sided Mann-Whitney *U*-test (right). Sixteen cell lines were divided into two groups of eight based on whether the measured variable (% $Mitf^{Low}/Axl^{Low}$ cells) had a value above or below the median. Data for each group was then presented using box-and-whisker plots, on which the central mark indicates the median, and the bottom and top edges of the box indicate the 25th and 75th percentiles, respectively. The whiskers extend to 1.5× the interquartile range as a measure of variance, and datapoints outside the range are plotted individually with asterisks. Source data are provided as a Source Data file.

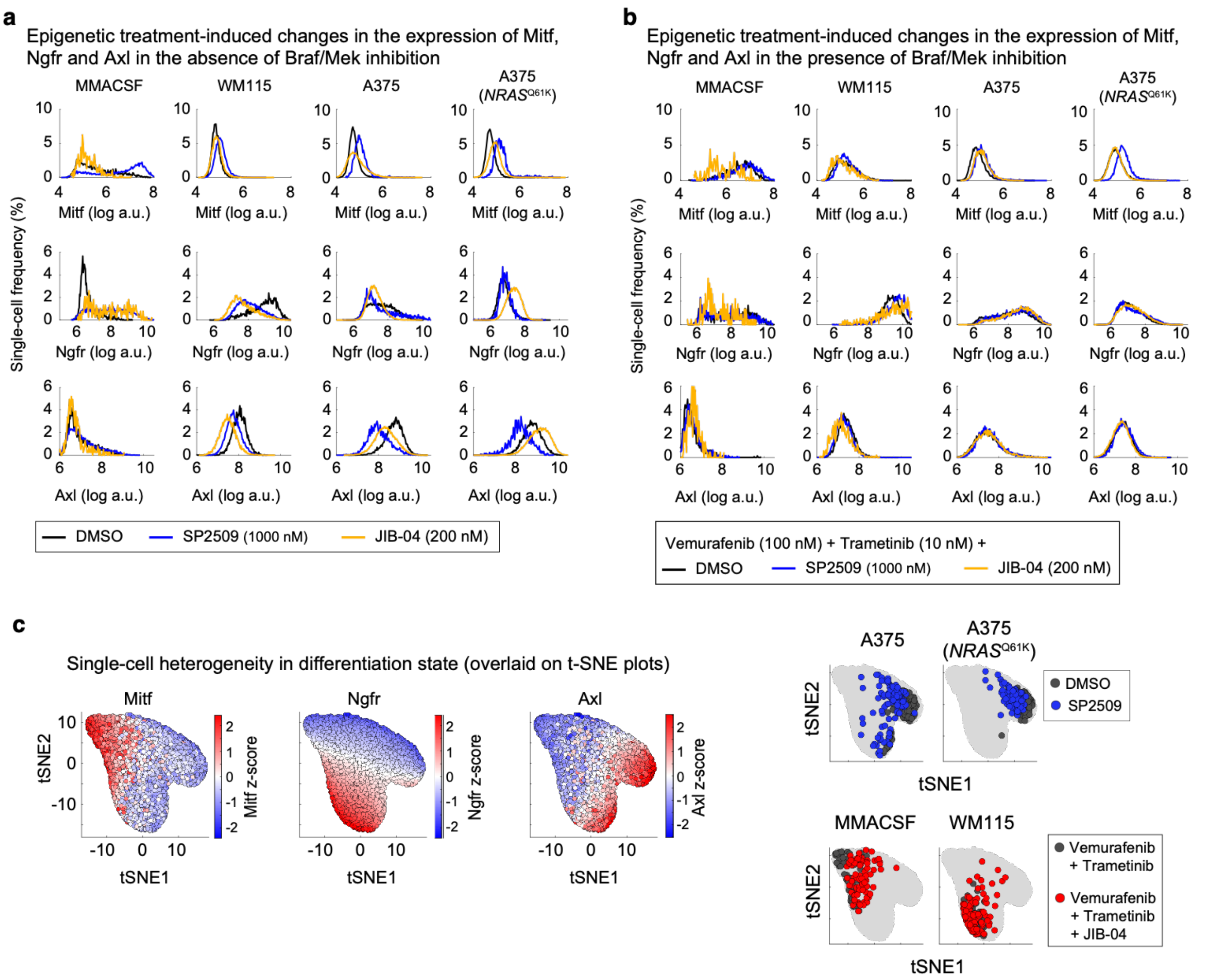

Supplementary Figure 18. Single-cell protein levels of Mitf (top), Ngfr (middle) and Axl (bottom), measured by immunofluorescence microscopy, in four melanoma cell lines following treatment with vehicle (DMSO), SP2509, or JIB-04 for 120 h in the absence of Braf/Mek inhibition **(a)** and in the presence of Braf/Mek inhibition **(b)** and overlaid on t-SNE visualization plots. Source data are provided as a Source Data file.

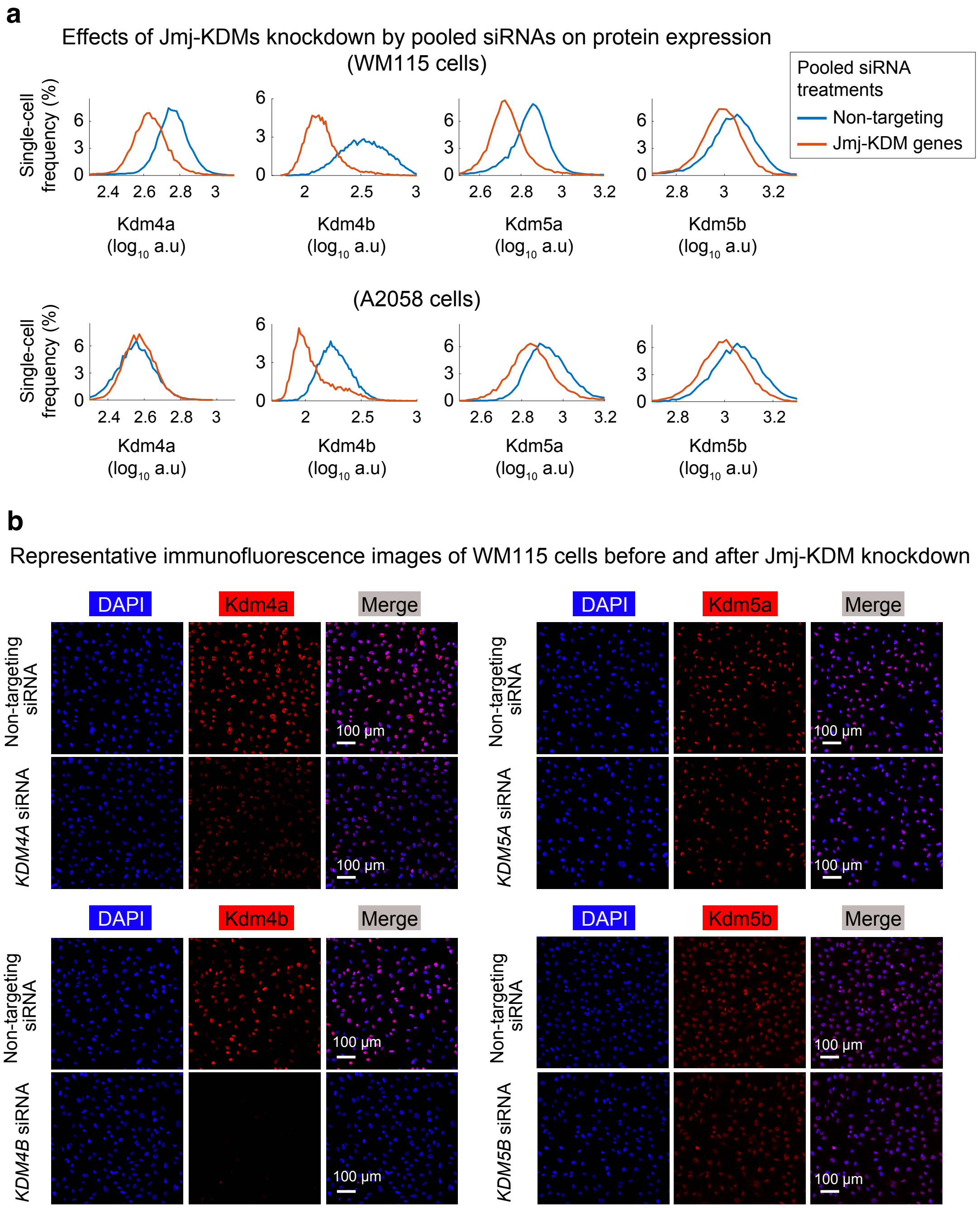

Supplementary Figure 19. Effects of Jmj-KDM knockdown on the corresponding protein expression levels at a single-cell level. (a) Single-cell protein levels of Jmj-KDM proteins Kdm4a, Kdm4b, Kdm5a, and Kdm5b, measured by immunofluorescence microscopy, in WM115 (top) and A2058 cells (bottom) following treatment with pooled siRNAs targeting either *KDM4A*, *KDM4B*, *KDM5A*, or *KDM5B*, or with non-targeting (control) siRNA for 96 h. **(b)** Representative immunofluorescence images of WM115 cells before and after each Jmj-KDM protein knockdown. Each experiment was repeated three times independently with similar result. Scale bars represent 100 µm. Source data are provided as a Source Data file.

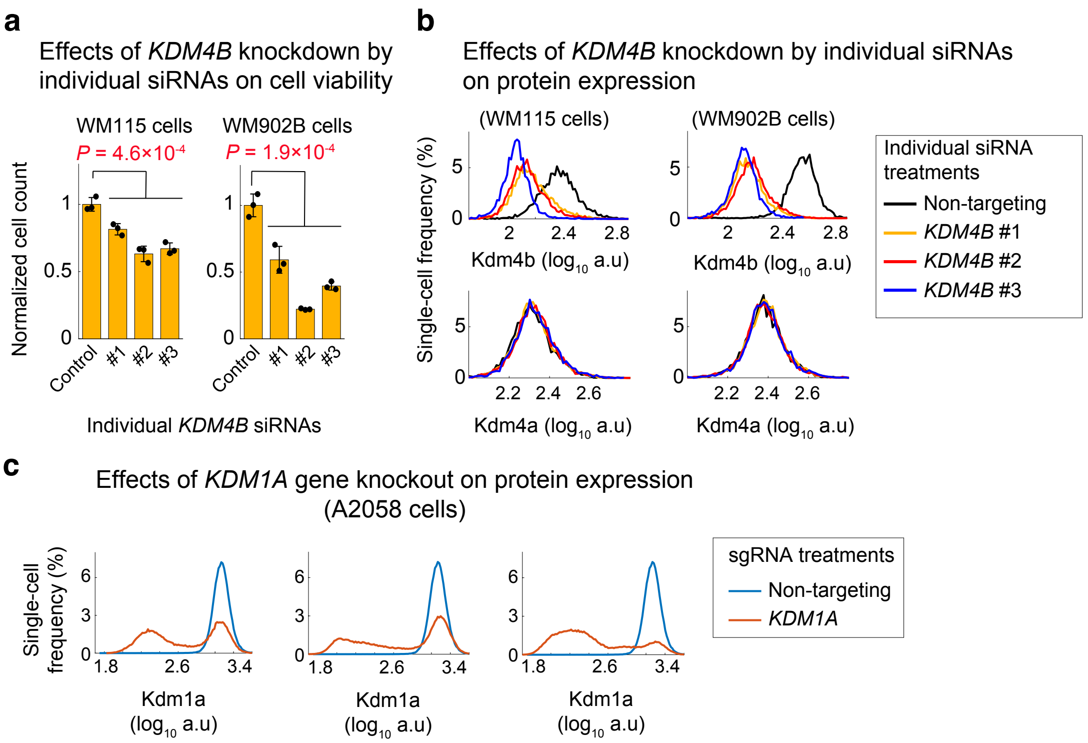

Supplementary Figure 20. Effects of *KDM4B* knockdown (by independent siRNAs) and *KDM1A* CRISPR knockout (by independent sgRNAs). (a) Relative cell viability in WM115 cells (left) and WM902B cells (right) following treatment with three independent siRNAs targeting *KDM4B* for 96 h. Viability data for each treatment condition were normalized to cells treated with non-targeting (control) siRNA. Data are presented as mean values ± s.d. calculated across n = 3 biologically independent experiments. Statistical significance was determined by two-sided *t* test. **(b)** Single-cell protein levels of Kdm4b (top) and Kdm4a (bottom), measured by immunofluorescence microscopy, in WM115 cells (left) and WM902B cells (right) following treatment with three independent siRNAs targeting *KDM4B* or with non-targeting (control) siRNA for 96 h. **(c)** Single-cell protein levels of Kdm1a, measured by immunofluorescence microscopy, in Cas9-positive A2058 cells following treatment with three different types of *KDM1A* lentiviral single guide RNA (sgRNA), or with non-targeting (control) sgRNA for 96 h. Source data are provided as a Source Data file.

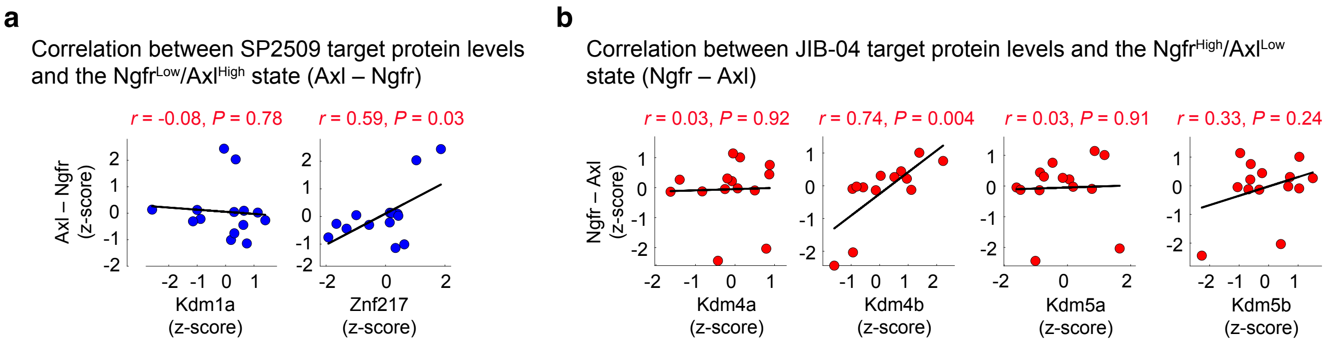

Supplementary Figure 21. Kdm4b and Znf217 protein levels are correlated with relative levels of melanoma differentiation state markers Ngfr and Axl. (a) Two-sided Pearson's correlation analysis of variations in the difference between Ngfr and Axl protein levels (Ngfr – Axl or Axl – Ngfr) and Kdm1a (left) or Znf217 (right) across *BRAF*-mutant melanoma cell lines. Protein data are extracted from the Cancer Cell Line Encyclopedia (CCLE) proteomics database (measured by multiplexed mass spectrometry) and z-scored across all of *BRAF*-mutant melanoma cell lines present in the database. **(b)** two-sided Pearson's correlation analysis of variations in the difference between Ngfr and Axl protein levels and JIB-04 targets, including Kdm4a, Kdm4b, Kdm5a and Kdm5b across *BRAF*-mutant melanoma cell lines. Protein data are extracted from the Cancer Cell Line Encyclopedia (CCLE) proteomics database (measured by multiplexed mass spectrometry) and z-scored across all of *BRAF*-mutant melanoma cell lines present in the database. Source data are provided as a Source Data file.

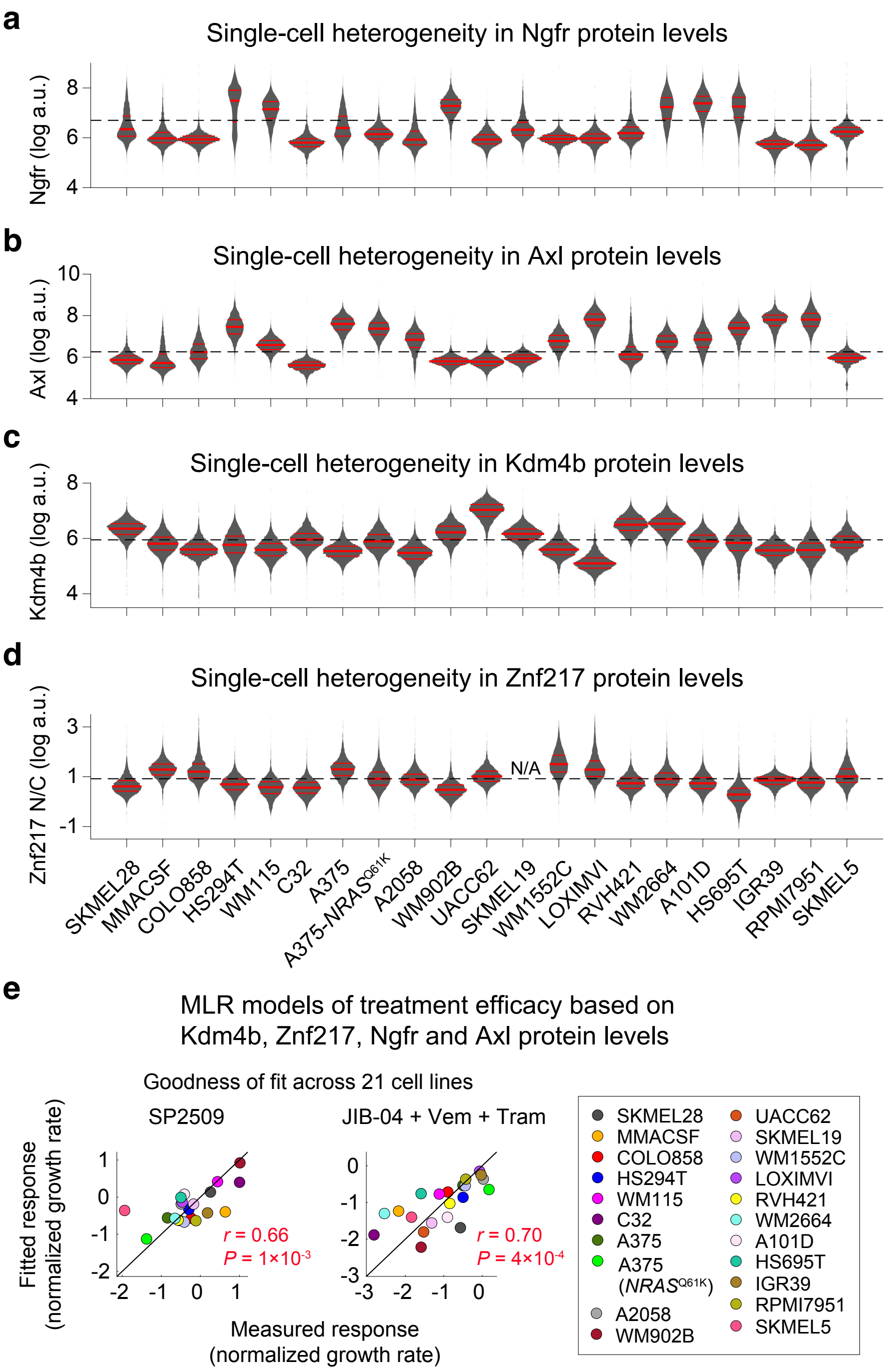

Supplementary Figure 22. Kdm4b and Znf217 protein levels predict differentiation state-specific sensitivity to JIB-04 and SP2509. (a-d) Single-cell heterogeneity in protein levels of Ngfr **(a)**, Axl **(b)**, Kdm4b **(c)** and Znf217 **(d)**, quantified by multiplexed immunofluorescence imaging across 21 melanoma cell lines. The distributions of single-cell data across different conditions are shown by violin plots, highlighting the median and interquartile (25% and 75%) ranges. **(e)** Two-sided Pearson's correlation between responses (normalized growth rates) to SP2509 (left) or JIB-04 in combination with vemurafenib and trametinib (right), measured for each of the 21 melanoma cell lines (x-axis) and corresponding responses fitted by multi-linear regression (MLR) analysis of all cell lines. Source data are provided as a Source Data file.

Supplementary Table 1. List of chemicals used in this study, including their sources and catalog numbers, nominal targets, and purity.

Vendor	Cat #	Compound	Nominal target	Purity
Selleck Chemicals	S1004	Veliparib (ABT-888)	PARP	99.72%
Selleck Chemicals	S1007	Roxadustat (FG-4592)	HIF	99.39%
Selleck Chemicals	S1030	Panobinostat (LBH589)	HDAC	99.51%
Selleck Chemicals	S1045	Trichostatin A (TSA)	HDAC	97.62%
Selleck Chemicals	S1047	Vorinostat (SAHA, MK0683)	Autophagy,HDAC	99.90%
Selleck Chemicals	S1048	Tozasertib (VX-680, MK-0457)	Aurora Kinase	99.88%
Selleck Chemicals	S1053	Entinostat (MS-275)	HDAC	99.96%
Selleck Chemicals	S1060	Olaparib (AZD2281, Ku-0059436)	PARP	99.70%
Selleck Chemicals	S1085	Belinostat (PXD101)	HDAC	99.74%
Selleck Chemicals	S1087	Iniparib (BSI-201)	PARP	99.80%
Selleck Chemicals	S1090	Abexinostat (PCI-24781)	HDAC	97.29%
Selleck Chemicals	S1095	Dacinostat (LAQ824)	HDAC	95.40%
Selleck Chemicals	S1096	Quisinostat (JNJ-26481585) 2HCl	HDAC	99.62%
Selleck Chemicals	S1098	Rucaparib (AG-014699,PF-01367338) phosphate	PARP	99.89%
Selleck Chemicals	S1100	MLN8054	Aurora Kinase	97.90%
Selleck Chemicals	S1103	ZM 447439	Aurora Kinase	96.53%
Selleck Chemicals	S1107	Danusertib (PHA-739358)	Aurora Kinase,Bcr-Abl,c-RET,FGFR	99.55%
Selleck Chemicals	S1122	Mocetinostat (MGCD0103)	HDAC	99.46%
Selleck Chemicals	S1129	SRT1720 HCl	Sirtuin	99.07%
Selleck Chemicals	S1132	INO-1001 (3-Aminobenzamide)	PARP	99.80%
Selleck Chemicals	S1133	Alisertib (MLN8237)	Aurora Kinase	99.64%
Selleck Chemicals	S1134	AT9283	Aurora Kinase,Bcr-Abl,JAK	100%
Selleck Chemicals	S1143	AG-490 (Tyrphostin B42)	EGFR,JAK	99.76%
Selleck Chemicals	S1147	Barasertib (AZD1152-HQPA)	Aurora Kinase	99.98%
Selleck Chemicals	S1154	SNS-314 Mesylate	Aurora Kinase	99.88%
Selleck Chemicals	S1168	Valproic acid sodium salt (Sodium valproate)	GABA Receptor,HDAC,Autophagy	100%
Selleck Chemicals	S1171	CYC116	Aurora Kinase,VEGFR	99.52%
Selleck Chemicals	S1181	ENMD-2076	Aurora Kinase,FLT3,VEGFR	99.41%
Selleck Chemicals	S1194	CUDC-101	EGFR,HDAC,HER2	99.36%
Selleck Chemicals	S1200	Decitabine	DNA Methyltransferase	99.93%
Selleck Chemicals	S1216	PFI-1 (PF-6405761)	Epigenetic Reader Domain	99.07%
Selleck Chemicals	S1233	2-Methoxyestradiol (2-MeOE2)	HIF	99.68%
Selleck Chemicals	S1249	JNJ-7706621	Aurora Kinase,CDK	99.45%
Selleck Chemicals	S1327	Ellagic acid	Topoisomerase	99.64%
Selleck Chemicals	S1378	Ruxolitinib (INCB018424)	JAK	99.92%
Selleck Chemicals	S1393	Pirarubicin	Topoisomerase	99.82%
Selleck Chemicals	S1396	Resveratrol	Autophagy,Sirtuin	99.89%
Selleck Chemicals	S1422	Droxinostat	HDAC	96.15%
Selleck Chemicals	S1451	Aurora A Inhibitor I	Aurora Kinase	99.40%
Selleck Chemicals	S1454	PHA-680632	Aurora Kinase	96.62%
Selleck Chemicals	S1463	Ofloxacin	Topoisomerase	99.91%
Selleck Chemicals	S1484	MC1568	HDAC	96.74%
Selleck Chemicals	S1509	Norfloxacin	Topoisomerase	100%
Selleck Chemicals	S1515	Pracinostat (SB939)	HDAC	99.77%
Selleck Chemicals	S1529	Hesperadin	Aurora Kinase	99.09%
Selleck Chemicals	S1541	Selisistat (EX 527)	Sirtuin	99.78%
Selleck Chemicals	S1782	Azacitidine	DNA Methyltransferase	99.92%
Selleck Chemicals	S2012	PCI-34051	HDAC	99.01%
Selleck Chemicals	S2018	ENMD-2076 L-(+)-Tartaric acid	Aurora Kinase,FLT3,VEGFR	99.36%
Selleck Chemicals	S2158	KW-2449	Aurora Kinase,Bcr-Abl,FLT3	99.72%
Selleck Chemicals	S2162	AZD1480	JAK	99.64%
Selleck Chemicals	S2170	Givinostat (ITF2357)	HDAC	99.07%
Selleck Chemicals	S2178	AG-14361	PARP	99.65%
Selleck Chemicals	S2179	Gandotinib (LY2784544)	JAK	99.60%
Selleck Chemicals	S2198	SGI-1776 free base	Pim	99.39%
Selleck Chemicals	S2214	AZ 960	JAK	99.05%
Selleck Chemicals	S2219	Momelotinib (CYT387)	JAK	99.08%
Selleck Chemicals	S2244	AR-42	HDAC	99.03%
Selleck Chemicals	S2391	Quercetin	Src,Sirtuin,PKC,PI3K	99.60%
Selleck Chemicals	S2554	Daphnetin	PKA,EGFR,PKC	99.23%
Selleck Chemicals	S2627	Tubastatin A HCl	HDAC	99.30%
Selleck Chemicals	S2686	NVP-BSK805 2HCl	JAK	98.32%
Selleck Chemicals	S2692	TG101209	c-RET,FLT3,JAK	99.74%
Selleck Chemicals	S2693	Resminostat	HDAC	99.04%
Selleck Chemicals	S2718	TAK-901	Aurora Kinase	99.05%
Selleck Chemicals	S2719	AMG-900	Aurora Kinase	99.03%
Selleck Chemicals	S2736	Fedratinib (SAR302503, TG101348)	JAK	99.96%

Selleck Chemicals	S2740	GSK1070916	Aurora Kinase	98.62%
Selleck Chemicals	S2759	CUDC-907	HDAC,PI3K	99.38%
Selleck Chemicals	S2770	MK-5108 (VX-689)	Aurora Kinase	98.40%
Selleck Chemicals	S2779	M344	HDAC	99.36%
Selleck Chemicals	S2789	Tofacitinib (CP-690550,Tasocitinib)	JAK	99.79%
Selleck Chemicals	S2796	WP1066	JAK	99.49%
Selleck Chemicals	S2804	Sirtinol	Sirtuin	97%
Selleck Chemicals	S2806	CEP-33779	JAK	99.56%
Selleck Chemicals	S2818	Tacedinaline (CI994)	HDAC	99.74%
Selleck Chemicals	S2821	RG108	DNA Methyltransferase,Transferase	99.74%
Selleck Chemicals	S2851	Baricitinib (LY3009104, INCB028050)	JAK	99.88%
Selleck Chemicals	S2867	WHI-P154	EGFR,JAK	99.02%
Selleck Chemicals	S2886	PJ34	PARP	99.25%
Selleck Chemicals	S2902	S-Ruxolitinib (INCB018424)	JAK	99.81%
Selleck Chemicals	S2919	IOX2	HIF	99.57%
Selleck Chemicals	S3001	Clevudine	DNA/RNA Synthesis	99.96%
Selleck Chemicals	S3147	Entacapone	Histone Methyltransferase	99.57%
Selleck Chemicals	S4125	Sodium Phenylbutyrate	HDAC	99.86%
Selleck Chemicals	S4246	Tranylcypromine (2-PCPA) HCl	MAO	99.84%
Selleck Chemicals	S4294	Procainamide HCl	DNA Methyltransferase,Sodium Channel	99.09%
Selleck Chemicals	S5001	Tofacitinib (CP-690550) Citrate	JAK	99.89%
Selleck Chemicals	S7029	AZD2461	PARP	99.29%
Selleck Chemicals	S7036	XL019	JAK	99.01%
Selleck Chemicals	S7041	CX-6258 HCl	Pim	99.41%
Selleck Chemicals	S7062	Pinometostat (EPZ5676)	Histone Methyltransferase	99.75%
Selleck Chemicals	S7070	GSK J4 HCl	Histone Demethylase	99.40%
Selleck Chemicals	S7079	SGC 0946	Histone Methyltransferase	99.49%
Selleck Chemicals	S7088	UNC1215	Epigenetic Reader Domain	99.73%
Selleck Chemicals	S7104	AZD1208	Pim	99.86%
Selleck Chemicals	S7110	(+)-JQ1	Epigenetic Reader Domain	99.84%
Selleck Chemicals	S7113	Zebularine	DNA Methyltransferase	99.67%
Selleck Chemicals	S7120	3-deazaneplanocin A (DZNeP) HCl	Histone Methyltransferase	99.81%
Selleck Chemicals	S7152	C646	Histone Acetyltransferase	98.73%
Selleck Chemicals	S7189	I-BET-762	Epigenetic Reader Domain	99.05%
Selleck Chemicals	S7229	RGFP966	HDAC	99.74%
Selleck Chemicals	S7231	GSK2801	Epigenetic Reader Domain	99.06%
Selleck Chemicals	S7233	Bromosporine	Epigenetic Reader Domain	99.92%
Selleck Chemicals	S7234	IOX1	Histone Demethylase	99.04%
Selleck Chemicals	S7237	OG-L002	Histone Demethylase	99.38%
Selleck Chemicals	S7238	NVP-TNKS656	Tankyrase	99.85%
Selleck Chemicals	S7256	SGC-CBP30	Epigenetic Reader Domain	99.11%
Selleck Chemicals	S7265	MM-102	Histone Methyltransferase	99.00%
Selleck Chemicals	S7276	SGI-1027	DNA Methyltransferase	99.58%
Selleck Chemicals	S7281	JIB-04	Jumonji histone demethylases	99.80%
Selleck Chemicals	S7292	RG2833 (RGFP109)	HDAC	99.17%
Selleck Chemicals	S7294	PFI-2 HCl	Histone Methyltransferase	99.05%
Selleck Chemicals	S7295	RVX-208	Epigenetic Reader Domain	99.47%
Selleck Chemicals	S7296	ML324	Histone Demethylase	99.02%
Selleck Chemicals	S7300	PJ34 HCl	PARP	99.25%
Selleck Chemicals	S7304	CPI-203	Epigenetic Reader Domain	99.69%
Selleck Chemicals	S7305	MS436	Epigenetic Reader Domain	95.98%
Selleck Chemicals	S7315	PFI-3	Epigenetic Reader Domain	99.87%
Selleck Chemicals	S7324	TMP269	HDAC	99.00%
Selleck Chemicals	S7353	EPZ004777	Histone Methyltransferase	99.09%
Selleck Chemicals	S7360	OTX015	Epigenetic Reader Domain	99.81%
Selleck Chemicals	S7373	UNC669	Epigenetic Reader Domain	99.07%
Selleck Chemicals	S7438	ME0328	PARP	99.89%
Selleck Chemicals	S7473	Nexturastat A	HDAC	99.65%
Selleck Chemicals	S7476	MG149	Histone Acetyltransferase	99.52%
Selleck Chemicals	S7541	Decernotinib (VX-509)	JAK	99.57%
Selleck Chemicals	S7555	4SC-202	HDAC	99.84%
Selleck Chemicals	S7570	UNC0379	Histone Methyltransferase	99.66%
Selleck Chemicals	S7572	A-366	Histone Methyltransferase	99.23%
Selleck Chemicals	S7574	GSK-LSD1 2HCl	Histone Demethylase KDM1A	99.78%
Selleck Chemicals	S7581	GSK J1	Histone Demethylase	99.67%
Selleck Chemicals	S7582	Anacardic Acid	Histone Acetyltransferase	99.72%
Selleck Chemicals	S7591	BRD4770	Histone Methyltransferase	99.75%
Selleck Chemicals	S7605	Filgotinib (GLPG0634)	JAK	99.72%
Selleck Chemicals	S7610	UNC0631	Histone Methyltransferase	99.35%
Selleck Chemicals	S7611	EI1	Histone Methyltransferase	99.04%

Selleck Chemicals	S7616	CPI-169	Histone Methyltransferase	99.00%
Selleck Chemicals	S7618	MI-2 (Menin-MLL Inhibitor)	Histone Methyltransferase	99.39%
Selleck Chemicals	S7619	MI-3 (Menin-MLL Inhibitor)	Histone Methyltransferase	99.03%
Selleck Chemicals	S7620	GSK1324726A (I-BET726)	Epigenetic Reader Domain	99.61%
Selleck Chemicals	S7625	Niraparib (MK-4827) tosylate	PARP	99.69%
Selleck Chemicals	S7641	Remodelin	Histone Acetyltransferase	99.39%
Selleck Chemicals	S7656	CPI-360	Histone Methyltransferase	99.72%
Selleck Chemicals	S7680	SP2509	Histone Demethylase KDM1A	99.05%
Selleck Chemicals	S7681	OF-1	Epigenetic Reader Domain	99.19%
Selleck Chemicals	S7748	EPZ015666(GSK3235025)	Histone Methyltransferase	>97%
Selleck Chemicals	S7767	AZ6102	Tankyrase	99.58%
Selleck Chemicals	S7795	ORY-1001 (RG-6016) 2HCl	Histone Demethylase KDM1A	99.95%
Selleck Chemicals	S7796	GSK2879552 2HCl	Histone Demethylase KDM1A	99.51%
Selleck Chemicals	S7804	GSK503	Histone Methyltransferase	99.84%
Selleck Chemicals	S7805	EPZ011989	Histone Methyltransferase	99.07%
Selleck Chemicals	S7832	SGC707	Histone Methyltransferase	99.77%
Selleck Chemicals	S7835	I-BRD9	Epigenetic Reader Domain	99.00%
Selleck Chemicals	S8001	Ricolinostat (ACY-1215)	HDAC	99.89%
Selleck Chemicals	S8004	ZM 39923 HCl	JAK	99.80%
Selleck Chemicals	S8005	SMI-4a	Pim	100.00%
Selleck Chemicals	S8006	BIX 01294	Histone Methyltransferase	100.00%
Selleck Chemicals	S8038	UPF 1069	PARP	99.47%
Selleck Chemicals	S8043	Scriptaid	HDAC	99.22%
Selleck Chemicals	S8049	Tubastatin A	HDAC	99.67%
Selleck Chemicals	S8056	Lomeguatrib	DNA Methyltransferase	99.37%
Selleck Chemicals	S8057	Pacritinib (SB1518)	FLT3,JAK	97.09%
Selleck Chemicals	S8096	Mirin	ATM/ATR	99.13%
Selleck Chemicals	S8111	GSK591	Histone Methyltransferase	99.00%
Selleck Chemicals	S8112	MS023	Histone Methyltransferase	99.15%
Selleck Chemicals	S8146	Mitomycin C	DNA/RNA Synthesis	99.74%
Selleck Chemicals	S8179	BI-7273	Epigenetic Reader Domain	99.79%
Selleck Chemicals	S8180	PF-CBP1 HCl	Epigenetic Reader Domain	99.01%
Selleck Chemicals	S8195	Oclacitinib	JAK	99.09%
Selleck Chemicals	S8209	HLCL-61 HCL	Histone Methyltransferase	99.79%
Selleck Chemicals	S8323	ITSA-1 (ITSA1)	HDAC	99.08%
Selleck Chemicals	S8481	SRT3025 HCl	Sirtuin	99.62%
Selleck Chemicals	S8496	EED226	Epigenetic Reader Domain	99.05%
Selleck Chemicals	S8502	TMP195	HDAC	99.05%
Selleck Chemicals	S8567	Tucidinostat (Chidamide)	HDAC	99.09%
Selleck Chemicals	S1055	Enzastaurin (LY317615)	PKC	99.47%
Selleck Chemicals	S1573	Fasudil (HA-1077) HCl	Autophagy,ROCK	100%
Selleck Chemicals	S1703	Divalproex Sodium	HDAC	99.91%
Selleck Chemicals	S1774	Thioguanine	DNA Methyltransferase	99.93%
Selleck Chemicals	S1802	AICAR (Acadesine)	AMPK	99.97%
Selleck Chemicals	S1848	Curcumin	NF-κB,HDAC,Histone Acetyltransferase,Nrf2	99.83%
Selleck Chemicals	S1899	Nicotinamide (Vitamin B3)	Sirtuin	100%
Selleck Chemicals	S2197	A-966492	PARP	99.80%
Selleck Chemicals	S2250	(-)-Epigallocatechin Gallate	DNA Methyltransferase,HER2,Telomerase,EGFR,Fatty Acid Synthase	99.68%
Selleck Chemicals	S2298	Fisetin	Sirtuin	97.76%
Selleck Chemicals	S2341	(-)-Parthenolide	HDAC,NF-κB,Mdm2,p53	100%
Selleck Chemicals	S2407	Curcumol	JAK	>95%
Selleck Chemicals	S2542	Phenformin HCl	AMPK	99.05%
Selleck Chemicals	S2697	A-769662	AMPK,Fatty Acid Synthase	99.12%
Selleck Chemicals	S2791	Sotrastaurin	PKC	99.57%
Selleck Chemicals	S2911	Go 6983	PKC	97.25%
Selleck Chemicals	S4170	Coumarin	Immunology & Inflammation related	99.97%
Selleck Chemicals	S4589	Amodiaquine dihydrochloride dihydrate	Transferase,Histone Methyltransferase	99.80%
Selleck Chemicals	S4710	Picolinamide	PARP	99.93%
Selleck Chemicals	S4715	Benzamide	PARP	98.15%
Selleck Chemicals	S4735	Salvianolic acid B	Sirtuin	99.73%
Selleck Chemicals	S4900	Tenovin-6	p53,Sirtuin	98.61%
Selleck Chemicals	S7065	MK-8745	Aurora Kinase	99.05%
Selleck Chemicals	S7119	Go6976	FLT3,JAK,PKC	99.34%
Selleck Chemicals	S7128	Tazemetostat (EPZ-6438)	Histone Methyltransferase	99.56%
Selleck Chemicals	S7144	BMS-911543	JAK	99.03%
Selleck Chemicals	S7165	UNC1999	Histone Methyltransferase	99.20%
Selleck Chemicals	S7207	Bisindolylmaleimide IX (Ro 31-8220 Mesylate)	PKC	99.02%
Selleck Chemicals	S7239	G007-LK	Tankyrase	99.00%
Selleck Chemicals	S7259	FLLL32	JAK	95.54%
Selleck Chemicals	S7278	HPOB	HDAC	99.36%

Selleck Chemicals	S7317	WZ4003	AMPK	98.00%
Selleck Chemicals	S7318	HTH-01-015	AMPK	99.07%
Selleck Chemicals	S7569	LMK-235	HDAC	99.05%
Selleck Chemicals	S7575	LLY-507	Histone Methyltransferase	97.33%
Selleck Chemicals	S7577	AGK2	Sirtuin	99.07%
Selleck Chemicals	S7588	Reversine	Adenosine Receptor,Aurora Kinase	96.15%
Selleck Chemicals	S7593	Splitomicin	HDAC	96.04%
Selleck Chemicals	S7595	Santacruzamate A (CAY10683)	HDAC	99.57%
Selleck Chemicals	S7596	CAY10603	HDAC	99.04%
Selleck Chemicals	S7612	PX-478 2HCl	HIF	>97%
Selleck Chemicals	S7617	Tasquinimod	HDAC	99.29%
Selleck Chemicals	S7634	Cerdulatinib (PRT062070, PRT2070)	JAK	97.82%
Selleck Chemicals	S7650	Peficitinib (ASP015K, JNJ-54781532)	JAK	99.67%
Selleck Chemicals	S7689	BG45	HDAC	97.91%
Selleck Chemicals	S7726	BRD73954	HDAC	99.65%
Selleck Chemicals	S7730	NU1025	PARP	99.29%
Selleck Chemicals	S7792	SRT2104 (GSK2245840)	Sirtuin	99.61%
Selleck Chemicals	S7815	MI-136	Histone Methyltransferase	99.05%
Selleck Chemicals	S7816	MI-463	Histone Methyltransferase	99.35%
Selleck Chemicals	S7817	MI-503	Histone Methyltransferase	99.16%
Selleck Chemicals	S7820	EPZ020411 2HCl	Histone Methyltransferase	99.08%
Selleck Chemicals	S7833	OICR-9429	Histone Methyltransferase	99.14%
Selleck Chemicals	S7843	BI-847325	MEK,Aurora Kinase	95.94%
Selleck Chemicals	S7845	SirReal2	Sirtuin	99.50%
Selleck Chemicals	S7853	CPI-0610	Epigenetic Reader Domain	99.95%
Selleck Chemicals	S7884	AMI-1	Histone Methyltransferase	99.02%
Selleck Chemicals	S7906	PFI-4	Epigenetic Reader Domain	99.74%
Selleck Chemicals	S7946	KC7F2	HIF	99.03%
Selleck Chemicals	S7953	ETC-1002	AMPK,LDL	99.13%
Selleck Chemicals	S7958	Lificiguat(YC-1)	HIF	99.94%
Selleck Chemicals	S7979	FG-2216	HIF	99.78%
Selleck Chemicals	S7983	A-196	Histone Methyltransferase	99.53%
Selleck Chemicals	S8000	Tenovin-1	E3 Ligase ,p53	99.82%
Selleck Chemicals	S8068	Chaetocin	Histone Methyltransferase	98.34%
Selleck Chemicals	S8071	UNC0638	Histone Methyltransferase	99.08%
Selleck Chemicals	S8138	Molidustat (BAY 85-3934)	HIF	99.82%
Selleck Chemicals	S8147	MS049	Histone Methyltransferase	99.48%
Selleck Chemicals	S8171	Daprodustat (GSK1278863)	HIF	99.29%
Selleck Chemicals	S8190	CPI-637	Epigenetic Reader Domain	99.61%
Selleck Chemicals	S8245	Thiomyristoyl	Sirtuin	99.84%
Selleck Chemicals	S8249	HPI-4 (Ciliobrevin A)	Hedgehog/Smoothened	99.63%
Selleck Chemicals	S8265	GSK6853	Epigenetic Reader Domain	99.44%
Selleck Chemicals	S8270	SRT2183	Sirtuin	99.19%
Selleck Chemicals	S8287	CPI-455 HCl	Histone Demethylase	99.08%
Selleck Chemicals	S8340	SGC2085	Histone Methyltransferase	99.05%
Selleck Chemicals	S8344	AZD5153	Epigenetic Reader Domain	98.19%
Selleck Chemicals	S8353	CPI-1205	Histone Methyltransferase	99.04%
Selleck Chemicals	S8359	UNC3866	Histone Methyltransferase	96.46%
Selleck Chemicals	S8363	NMS-P118	PARP	99.90%
Selleck Chemicals	S8370	BGP-15 2HCl	PARP	99.94%
Selleck Chemicals	S8400	Mivebresib(ABBV-075)	Epigenetic Reader Domain	99.23%
Selleck Chemicals	S8419	E7449	PARP	97.48%
Selleck Chemicals	S8429	PNU-74654	Wnt/beta-catenin	99.49%
Selleck Chemicals	S8441	LW 6	HIF	99.00%
Selleck Chemicals	S8443	MK-8617	HIF	99.39%
Selleck Chemicals	S8460	Salermide	Sirtuin	99.79%
Selleck Chemicals	S8464	Citarinostat (ACY-241)	HDAC	99.03%
Selleck Chemicals	S1149	Gemcitabine HCl	Autophagy,DNA/RNA Synthesis	99.96%
Selleck Chemicals	S1215	Carboplatin	DNA/RNA Synthesis	99.26%
Selleck Chemicals	S1373	Daptomycin	DNA/RNA Synthesis	99.30%
Selleck Chemicals	S1384	Mizoribine	DNA/RNA Synthesis	99.96%
Selleck Chemicals	S1648	Cytarabine	DNA/RNA Synthesis	99.98%
Selleck Chemicals	S1826	Nedaplatin	DNA/RNA Synthesis	99.74%
Selleck Chemicals	S1995	Procarbazine HCl	DNA/RNA Synthesis	99.01%
Selleck Chemicals	S7419	Blasticidin S HCl	DNA/RNA Synthesis	99.91%
Selleck Chemicals	S8197	APTSTAT3-9R	STAT	99.56%
Selleck Chemicals	S1950	Metformin HCl	Autophagy	99.87%
Selleck Chemicals	S1999	Sodium butyrate	HDAC	100%
Selleck Chemicals	S7306	Dorsomorphin (Compound C) 2HCl	AMPK	99.67%
Selleck Chemicals	S1267	Vemurafenib	BRAF	99.26%

Selleck Chemicals	S2673	Trametinib	MEK	99.65%
Medchem Express	HY-103713	Seclidemstat (SP-2577)	Histone Demethylase KDM1A	98.78%

Supplementary Table 2. Small molecule screening data

Category	Parameter	Description
Assay	Type of assay	In vitro phenotypic profiling of small molecule epigenetic-modifying compound's effects (individually or in combination with Braf/Mek inhibitors) on melanoma cells
	Target	Epigenetic modifiers
	Primary measurement	Melanoma cell count (by DAPI) using fluorescence microscopy
	Key reagents	See chemical reagent table and methods
	Assay protocol	"Epigenetic compound screen" and "Immunofluorescence staining, quantitation and analysis" in manuscript
	Additional comments	
Library	Library size	276
	Library composition	Epigenetic-modifying compounds
	Source	Selleck Chemicals
	Additional comments	
Screen	Format	96-well plates
	Concentration(s) tested	0.2 µM and 1 µM for all compounds; equivalent volume of DMSO or water as vehicle control
	Plate controls	DMSO or water
	Reagent/ compound dispensing system	HP D300e Digital Dispenser
	Detection instrument and software	ImageXpress Micro Confocal High-Content Imaging System (Molecular Devices) and MetaXpress Imaging software, version 6.2.3.733
	Assay validation/QC	Replicate $R^2 > 0.98$
	Correction factors	
	Normalization	Growth rate, Mitf, p-Rb and p-Erk levels for cells treated with each epigenetic compound were normalized to cells treated with the vehicle (DMSO or water).
	Additional comments	
Post-HTS analysis	Hit criteria	A statistically significant decrease in normalized growth rate of cells treated with the epigenetic compound in at least one of the two cell lines and at least one of the tested MAPK inhibitor conditions (vemurafenib, vemurafenib plus trametinib or DMSO vehicle)
	Hit rate	58/276 ≈ 21%
	Additional assay(s)	Immunofluorescence microscopy of cell signaling (p-Erk), differentiation state (Mitf) and proliferation marker (p-Rb)
	Confirmation of hit purity and structure	N/A
	Additional comments	Screen data made available

Supplementary Table 3. Sequences of siRNAs and sgRNAs used in this study.

Gene siRNA/sgRNA	Vendor	Cat #	Sequence
KDM4A siRNA	Dharmacon	J-004292-05	GUAUGAUCUUCCAGACUUA
KDM4A siRNA	Dharmacon	J-004292-06	GCACGGACAUCAACCUUUC
KDM4A siRNA	Dharmacon	J-004292-07	GGGAUUCUAUCUCUUCUGA
KDM4A siRNA	Dharmacon	J-004292-08	GUGCGGAGUCUACCAAUUU
KDM4B siRNA	Dharmacon	J-004290-08	CCGCAGGUCUCACCGGAAA
KDM4B siRNA	Dharmacon	J-004290-09	GCGCAGAAUCUACCAACUU
KDM4B siRNA	Dharmacon	J-004290-10	CAAAUACGUGGCCUACAUA
KDM4B siRNA	Dharmacon	J-004290-11	CAUCAGCGGCUCUUUGUAU
KDM5A siRNA	Dharmacon	J-003297-22	GCAAAUGAGACAACGGAAA
KDM5A siRNA	Dharmacon	J-003297-23	GAACAGGCGGCUCGAGAAA
KDM5A siRNA	Dharmacon	J-003297-24	UGACAAUGGUGGACCGCAU
KDM5A siRNA	Dharmacon	J-003297-25	GGAUGAACAUUCUGCCGAA
KDM5B siRNA	Dharmacon	J-009899-05	GGAGAUGCACUUCGAUAUA
KDM5B siRNA	Dharmacon	J-009899-06	UAAGUUAGUUGCAGAAGAA
KDM5B siRNA	Dharmacon	J-009899-07	UCGAAGAGAUCCCUGCAUA
KDM5B siRNA	Dharmacon	J-009899-08	GGAAGAUCUUGGACUUAUU
KDM1A sgRNA	Dharmacon	VSGH10142-246522030	AGTGCGACAGGTTCGCTACA
KDM1A sgRNA	Dharmacon	VSGH10142-246999191	ATGTATACCACACCTTGCAT
KDM1A sgRNA	Dharmacon	VSGH10142-246522028	GTAGGAGGTCCTTACTTGGT
