# Supplementary material for: Epigenetic modulation reveals differentiation state specificity of oncogene addiction
Source: Nat Commun. 2021 Mar 9;12:1536. doi: 10.1038/s41467-021-21784-2 (PMC7943789; doi:10.1038/s41467-021-21784-2)
Supplement: Supplementary file 3 — Reporting Summary [file 41467_2021_21784_MOESM3_ESM.pdf]

nature research

Corresponding author(s): Mohammad Fallahi-Sichani

Last updated by author(s): Feb 10, 2021

Reporting Summary

Nature Research wishes to improve the reproducibility of the work that we publish. This form provides structure for consistency and transparency in reporting. For further information on Nature Research policies, see our Editorial Policies and the Editorial Policy Checklist.

Statistics

For all statistical analyses, confirm that the following items are present in the figure legend, table legend, main text, or Methods section.

n/a	Confirmed	
☐	☒	The exact sample size (*n*) for each experimental group/condition, given as a discrete number and unit of measurement
☐	☒	A statement on whether measurements were taken from distinct samples or whether the same sample was measured repeatedly
☐	☒	The statistical test(s) used AND whether they are one- or two-sided<br>*Only common tests should be described solely by name; describe more complex techniques in the Methods section.*
☐	☒	A description of all covariates tested
☐	☒	A description of any assumptions or corrections, such as tests of normality and adjustment for multiple comparisons
☐	☒	A full description of the statistical parameters including central tendency (e.g. means) or other basic estimates (e.g. regression coefficient) AND variation (e.g. standard deviation) or associated estimates of uncertainty (e.g. confidence intervals)
☐	☒	For null hypothesis testing, the test statistic (e.g. *F*, *t*, *r*) with confidence intervals, effect sizes, degrees of freedom and *P* value noted<br>*Give P values as exact values whenever suitable.*
☒	☐	For Bayesian analysis, information on the choice of priors and Markov chain Monte Carlo settings
☐	☒	For hierarchical and complex designs, identification of the appropriate level for tests and full reporting of outcomes
☐	☒	Estimates of effect sizes (e.g. Cohen's *d*, Pearson's *r*), indicating how they were calculated

Our web collection on statistics for biologists contains articles on many of the points above.

Software and code

Policy information about availability of computer code

Data collection	All microscopy data were collected using an ImageXpress Micro Confocal High-Content Imaging System (Molecular Devices) or Operetta CLS High-Content Imaging System (Perkin Elmer) and the associated built-in software.
Data analysis	Quantitative analysis of the microscopy images was performed using ImageJ version 2.1.0 (for background subtraction) and CellProfiler version 3.1.9 (for image segmentation and signal intensity quantification). All statistical analyses were performed using built-in functions of MATLAB versions 2018b and 2019b. Custom MATLAB scripts for the analysis of drug response, drug combination interactions based on the Bliss Independence model, t-SNE, PLSR and MLR analysis are available on GitHub at the following address: https://github.com/fallahi-sichani-lab/epigeneticModulationAnalysis.

For manuscripts utilizing custom algorithms or software that are central to the research but not yet described in published literature, software must be made available to editors and reviewers. We strongly encourage code deposition in a community repository (e.g. GitHub). See the Nature Research guidelines for submitting code & software for further information.

Data

Policy information about availability of data

All manuscripts must include a data availability statement. This statement should provide the following information, where applicable:

- Accession codes, unique identifiers, or web links for publicly available datasets
- A list of figures that have associated raw data
- A description of any restrictions on data availability

All data generated in this study are included in this published article and its supplementary information files. Source data for all figures are provided with the paper. Because of the large number and size of raw immunofluorescence microscopy images associated with this study, all relevant raw image files will be made available by hard drive upon reasonable request to the corresponding author. The list of of figures that have associated raw data (included in the Source Data file) is as

follows: Figures 1-6, Supplementary Figures 1, 3-6, 8-22. These include the raw data for all high-throughput experiments, including growth rate inhibition assays, epigenetic compound screening, single-cell profiling of MAPK signaling and differentiation state in melanoma cells, as well as profiling of signaling and phenotypic responses to epigenetic inhibitors are presented in the Source Data file. The Cancer Cell Line Encyclopedia (CCLE) mass spectrometry-based proteomics data were downloaded from the depmap project portal (https://depmap.org/portal/download/).

Field-specific reporting

Please select the one below that is the best fit for your research. If you are not sure, read the appropriate sections before making your selection.

- [x] Life sciences
- [ ] Behavioural & social sciences
- [ ] Ecological, evolutionary & environmental sciences

For a reference copy of the document with all sections, see nature.com/documents/nr-reporting-summary-flat.pdf

Life sciences study design

All studies must disclose on these points even when the disclosure is negative.

Sample size

No statistical method was used to predetermine sample size. Sample sizes were chosen based on similar studies in the relevant literature in order to unravel meaningful conclusions and according to the throughput and dimensionality of experimental measurements (across cell types, dose and time of drug treatments and the number of cell lines tested). The description of the rationale for the choice of sample sizes for each experiment is as follows.

1) For the first stage of the epigenetic compound screen which was performed in 2 cell lines, treated with 2 doses of each of the 276 epigenetic compounds (individually or in combination with MAPK inhibitor conditions vemurafenib, or vemurafenib plus trametinib) at 3 time-points, a sample size of n = 2 was used for each condition involving an epigenetic compound. This sample size was sufficient, because the primary purpose of the first stage of screen was to identify any compound that may cause a change in cell numbers in "at least" one of the tested conditions (i.e. cell lines, time-points, or MAPK inhibitor conditions) compared to cells that were not treated with any epigenetic compound. Statistical significance was evaluated based on $P < 0.05$ using two-sided t-test following correction for multiple comparisons using the Dunn-Sidak method, leading to the identification of 58 compounds for the follow-up reconfirmation analysis in the second stage of the compound screen. In addition, the correlation between cell count measurements across 2 replicates were evaluated (two-sided Pearson's $r > 0.99$, P value ~ 0) and unsupervised clustering of the normalized cell counts and protein measurements across 2 replicates revealed remarkable similarities among classes of compounds with common nominal epigenetic targets, which further demonstrated that a sample size of n = 2 was sufficient for the epigenetic compound screen.

2) For the follow-up cell count experiments using the selected epigenetic compounds across 21 melanoma cell lines, all measurements were performed at 3 time-points. In these experiments, the primary goal was to identify the growth rate of cells in response to each treatment condition rather than to compare cell counts. Therefore, we chose a sample size of n = 2, which across 3 time-points led to a total 6 data-points that are sufficient for the estimation of growth rates, i.e. the slope of the line that fits the log-normalized cell count data.

3) For the follow-up protein measurement experiments using the selected epigenetic compounds across 8 melanoma cell lines, all measurements were performed at 3 time-points in response to different drugs and combinations of drugs. In these experiments, the primary goal was to identify statistically significant patterns of correlation across responses of cells to different drugs using partial least square regression (PLSR) analysis, rather than to compare measurements with one another. Therefore, we chose a sample size of n = 2, which across 3 time-points, 24 treatment conditions, and 8 cell lines led to a total 1152 data-points. Using these data, PLSR analysis identified predictive models of drug response, which were evaluated statistically by cross-validation. Model predictions were also validated through independent experiments. Therefore, a sample size of n = 2 was appropriate for these high-throughput experiments.

4) For immunostaining experiments following specific gene knockdown or knockout studies, a sample size of n = 3 or n = 4 was used and the P value was reported using two-sided t-test. We determined this to be sufficient owing to internal control (specific staining of samples using known protein markers) and low observed variability between stained samples.

Data exclusions

No data were excluded.

Replication

All data were replicated using at least two biologically independent experiments. To assure the reproducibility of our findings regarding the efficacy of the epigenetic compounds, the compound screening was performed in three stages, where results on selected compounds from each stage were confirmed using a selected number of compounds in a follow-up stage. In addition, the robustness of the experimental findings (collected from an initial group of 9 cell lines) were tested using additional cell lines (up to 21). Because the screen was performed across a large number of multi-well plates assayed during a period of a few months, specific conditions that did not include an epigenetic compound treatment were repeated in all plates during the entire period of compound screening, creating a total of 276 replicates that were used to evaluate plate-to-plate and day-to-day robustness.

Randomization

Drugs and doses were not randomized in multi-well plates. Compounds that had the same vehicle (DMSO versus water) were tested together, because of the technical need of the drug dispenser. Because the screen was performed across a large number of multi-well plates assayed during a period of a few months, specific conditions that did not include an epigenetic compound treatment were repeated in all plates during the entire period of compound screening, creating a total of 276 replicates that were used to evaluate plate-to-plate and day-to-day robustness. For animal experiments, the mice were randomly allocated to two treatment groups (drug treatment versus vehicle).

Blinding

Blinding was generally not performed for this study, as the investigators had no prior assumption/bias regarding the expected outcomes. This is mostly because the findings are the result of an unbiased screen of a compound library.

Reporting for specific materials, systems and methods

We require information from authors about some types of materials, experimental systems and methods used in many studies. Here, indicate whether each material, system or method listed is relevant to your study. If you are not sure if a list item applies to your research, read the appropriate section before selecting a response.

Materials & experimental systems

n/a	Involved in the study
	☒ Antibodies
	☒ Eukaryotic cell lines
☒	Palaeontology and archaeology
	☒ Animals and other organisms
☒	Human research participants
☒	Clinical data
☒	Dual use research of concern

Methods

n/a	Involved in the study
☒	ChIP-seq
☒	Flow cytometry
☒	MRI-based neuroimaging

Antibodies

Antibodies used	The following primary monoclonal antibodies (mAb, clone name) and polyclonal antibodies (pAb) with specified animal sources, catalog numbers, research resource identifiers (RRID), and dilution ratios, were used in immunofluorescence staining assays: MITF (mouse mAb, clone D5, Abcam, Cat# ab3201, AB_303601, 1:800), p-ERKT202/Y204 (rabbit mAb, clone D13.14.4E, Cell Signaling Technology, Cat# 4370, AB_2315112, 1:800), Ki-67 (mouse mAb, clone 8D5, Cell Signaling Technology, Cat# 9449, AB_2715512, 1:1200), AXL (goat pAb, R&D Systems, Cat# AF154, AB_354852, 1:400), p-RbS807/811 (goat pAb, Santa Cruz Biotechnology, Cat# sc-16670, AB_655250, 1:400), NGFR (rabbit mAb, clone D4B3, Cell Signaling Technology, Cat# 8238, AB_10839265, 1:1600), p-S6S235/S236 (rabbit mAb, clone D57.2.2E, Cell Signaling Technology, Cat# 4851, AB_10695457, 1:400), SOX10 (mouse mAb, clone SOX10/991, Abcam, Cat# ab212843, AB_2889150, 1:1200), p-H2A.XS139 (rabbit mAb, clone EP854(2)Y, Abcam, Cat# ab195188, AB_2889151, 1:700), KDM1A (rabbit mAb, clone C69G12, Cell Signaling Technology, Cat# 2184, AB_2070132, 1:1600), KDM4A (rabbit mAb, clone C37E5, Cell Signaling Technology, Cat# 5328, AB_10828595, 1:100), KDM4B (rabbit mAb, clone D7E6, Cell Signaling Technology, Cat# 8639, AB_11140642, 1:100), KDM5A (rabbit mAb, clone EPR18651, Abcam, Cat# ab194286, AB_2889152, 1:1600), KDM5B (rabbit mAb, clone EPR12704, Abcam, Cat# ab181089, AB_2889153, 1:100), and ZNF217 (rabbit pAb, Thermo Fisher Scientific, Cat# 720352, AB_2716919, 1:200). The following secondary antibodies with specified sources and catalog numbers were used at a 1:2000 dilution: anti-rabbit Alexa Fluor 488 (Thermo Fisher, Cat# A21206, AB_2535792), anti-mouse Alexa Fluor 647 (Thermo Fisher, Cat# A31571, AB_162542), anti-goat Alexa Fluor 568 (Thermo Fisher, Cat# A11057, AB_2534104), anti-mouse Alexa Fluor 568 (Thermo Fisher, Cat# A10037, AB_2534013), and anti-rabbit Alexa Fluor 647 (Thermo Fisher, Cat# A31573, AB_2536183).
Validation	All of the antibodies used in this study were provided by commercial sources (Abcam, Cell Signaling Technology, Santa Cruz Biotechnology, and Thermo Fisher). Primary antibodies have been validated for the species and application by the manufacturer. The manufacturer's validation statements for the immunofluorescence application (used in this study) are described below:<br>Abcam: https://www.abcam.com/primary-antibodies/how-we-validate-our-antibodies#IHC%20and%20ICC<br>Cell Signaling Technology: https://www.cellsignal.com/about-us/our-approach-process/antibody-validation-immunofluorescence<br>Thermo Fisher: https://www.thermofisher.com/us/en/home/life-science/antibodies/invitrogen-antibody-validation.html<br>The Research Resource Identifiers (RRIDs) for all antibodies used in this study are provided above. The antibody RRID can be looked up in the Antibody Registry (https://antibodyregistry.org/) for further comments regarding the validation of each antibody with associated references from the literature. In our experiments, appropriate dilutions and staining conditions were further optimized for each specific antibody using appropriate positive and negative controls, e.g. by using specific cell lines with known target expression levels.

Eukaryotic cell lines

Policy information about cell lines

Cell line source(s)	BRAF-mutant melanoma cell lines used in this study were obtained from the following sources: COLO858 (from ECACC), RVH421 (from DSMZ), A375, A375(NRASQ61K), C32, A2058, WM115, SKMEL28, HS294T, WM1552C, HS695T, RPMI7951, SKMEL5, A101D, IGR39, and human adult primary epidermal melanocytes (all from ATCC), LOXIMV1 (from DCTD Tumor Repository, National Cancer Institute), MMACSF (RIKEN BioResource Center), WM902B and WM2664 (from Wistar Institute), UACC62 and SKMEL19 (from the Cancer Cell Line Encyclopedia).
Authentication	All of the cell lines have been periodically subjected to re-confirmation by Short Tandem Repeat (STR) profiling by ATCC.
Mycoplasma contamination	All cell lines are periodically tested by MycoAlertTM PLUS mycoplasma detection Kit (Lonza), and they all have been free of any Mycoplasma contamination.
Commonly misidentified lines (See ICLAC register)	No commonly misidentified cell line was used.

Animals and other organisms

Policy information about studies involving animals; ARRIVE guidelines recommended for reporting animal research

Laboratory animals	Athymic, 5-6 weeks old female nude (NU/J) mice were purchased from The Jackson Laboratory and used in this study.
Wild animals	Study did not involve wild animals.
Field-collected samples	Study did not involve samples collected from the filed.

Ethics oversight	All mouse experiments were carried out in accordance with procedures approved by the Institutional Animal Care and Use Committee (IACUC) at the University of Michigan.

Note that full information on the approval of the study protocol must also be provided in the manuscript.
