# Supplementary material for: Epigenetic modulation reveals differentiation state specificity of oncogene addiction
Source: Nat Commun. 2021 Mar 9;12:1536. doi: 10.1038/s41467-021-21784-2 (PMC7943789; doi:10.1038/s41467-021-21784-2)
Supplement: Supplementary file 2 — Peer Review File [file 41467_2021_21784_MOESM2_ESM.pdf]

Editorial Note: This manuscript has been previously reviewed at another journal that is not operating a transparent peer review scheme. The manuscript was considered suitable for publication without further review at *Nature Communications*.
